# Machine Learning Models to Predict Risk of Maternal Morbidity and Mortality From Electronic Medical Record Data: Scoping Review

**DOI:** 10.2196/68225

**Published:** 2025-08-14

**Authors:** Lavanya Vasudevan, Mohammad Golam Kibria, Lauren M Kucirka, Karl Shieh, Mian Wei, Safoora Masoumi, Subha Balasubramanian, Ashley Victor, Jamie L Conklin, Metin Nafi Gurcan, Alison M Stuebe, David Page

**Affiliations:** 1 Hubert Department of Global Health, Rollins School of Public Health, Emory University, 1518 Clifton Rd NE, Atlanta, GA, 30322, United States, 1 404-727-8812; 2 Duke Global Health Institute, Duke University, Durham, NC, United States; 3 Carolina Health Informatics Program, University of North Carolina at Chapel Hill, Chapel Hill, NC, United States; 4 Department of Obstetrics and Gynecology, School of Medicine, University of North Carolina at Chapel Hill, Chapel Hill, NC, United States; 5 School of Data Science and Society, University of North Carolina at Chapel Hill, Chapel Hill, NC, United States; 6 Department of Biostatistics and Bioinformatics, Duke University, Durham, NC, United States; 7 Department of Epidemiology, Gillings School of Public Health, University of North Carolina at Chapel Hill, Chapel Hill, NC, United States; 8 Health Sciences Library, University of North Carolina at Chapel Hill, Chapel Hill, NC, United States; 9 Center for Artificial Intelligence Research, School of Medicine, Wake Forest University, Winston-Salem, NC, United States; 10 Department of Maternal and Child Health, Gillings School of Global Public Health, University of North Carolina at Chapel Hill, Chapel Hill, NC, United States

**Keywords:** pregnancy, machine learning, maternal health, electronic medical records, maternal morbidity, maternal mortality, EHR, ML, scoping review, electronic health records, maternal death, EMR, delivery outcomes, cardiovascular risks, hypertensive disorder, gestational diabetes, post-partum hemorrhage, risk prediction

## Abstract

**Background:**

A majority (>80%) of maternal deaths in the United States are preventable. Using machine learning (ML) models that are generated from electronic medical records (EMRs) may be a promising approach to predict the risk of adverse maternal outcomes and enable proactive intervention to prevent maternal mortality. Current evidence syntheses of such ML approaches either focus only on specific maternal outcomes, aspects other than risk prediction, or do not consider the full pipeline of studies from the development to implementation in clinical practice.

**Objective:**

The goal of this scoping review is to document evidence for the use of ML models for predicting the risk of maternal morbidity and mortality outcomes (research objective [RO1]), the translation of such models into applications for clinical use by providers (RO2), and factors associated with the implementation of clinical applications in practice (RO3).

**Methods:**

The review was limited to studies in health care settings, using data from EMRs. A detailed search string was developed in collaboration with a health sciences librarian and implemented on February 20, 2023, on PubMed, CINAHL Plus, Scopus, Embase, and IEEE Xplore. Two reviewers independently reviewed titles and abstracts for inclusion, and a third reviewer resolved conflicts. Only full-length journal articles published in English were included. Studies using non-EMR data exclusively were excluded. Two reviewers independently reviewed full texts for inclusion, and a third reviewer resolved conflicts. A structured template was used for data extraction, and findings were summarized descriptively.

**Results:**

From 480 deduplicated studies identified from the search, 142 studies were included for full-text review, and 39 studies were included in the review. More than half of the included studies were conducted in 2022, and 34 studies were from just 3 countries (United States, China, and Israel). More studies focused on identifying the risk of pregnancy and delivery outcomes compared with postpartum outcomes. The top 3 most common outcomes for risk prediction were cardiovascular risks and hypertensive disorders of pregnancy (9 studies), gestational diabetes (7 studies), and postpartum hemorrhage (6 studies). Data were labeled with computable phenotypes in 30 studies, and the most often used method in ML models was boosting methods (18 studies). The most common metric used to assess model performance was area under the precision-recall curve (AUPRC; 33 studies). No studies described clinical applications of ML models for providers (RO2) or associated implementation factors (RO3).

**Conclusions:**

Key recommendations for future research and practice include expanding efforts to study maternal morbidity and mortality outcomes in the postpartum period, increasing transparency and reproducibility of studies through use of reporting checklists, and expanding efforts to implement ML models in clinical practice.

## Introduction

Maternal mortality is higher in the United States compared with other high-income nations, with 22.3 deaths per 100,000 live births in 2022 compared with 0, 3.5, and 8.4 deaths in Norway, Germany, and Canada, respectively [1,2]. In addition, there are profound racial disparities, with maternal mortality risk estimated to be 3-fold higher among individuals identifying as Black in comparison with those identifying as White [3]. The mechanisms underlying these disparities are likely multifaceted, including differences in access to care, quality of care, prevalence of chronic diseases, and the impact of implicit bias and structural racism [4-9]. Data collected and analyzed by Maternal Mortality Review Committees suggest that >80% of pregnancy-related deaths in the United States are preventable, underscoring the critical importance of developing interventions to reduce both the overall rates and large disparities in mortality that have been reported [10].

Maternal mortality is commonly referred to as “the tip of the iceberg” because for every maternal death, there are many more individuals who endure outcomes that portend long-term adverse health consequences [11-14]. The increased use of electronic medical record (EMR) systems has produced vast volumes of structured and unstructured health care data, offering opportunities for early intervention using machine learning (ML) models that identify individuals at risk of maternal morbidity and mortality [15,16]. ML methods recognize rules and patterns in data to generate predictive models and can handle nonlinear problems that generally arise in human physiology owing to intricate interactions among social drivers of health, and clinical and biological features [17]. ML models have been tested for accuracy in predicting adverse pregnancy outcomes before they happen [18]. For instance, deep learning–based or hybrid models, which are sophisticated and complex methods that can handle both structured and unstructured medical data, including diagnosis results, often offer good prediction accuracy [19]. In addition, ML approaches have been proposed to supplement clinician awareness of high-risk cases, to diagnose or forecast clinically relevant events, and facilitate improved clinical decision-making [20]. Despite the tremendous potential and significant investments, there are few real-world implementations of ML-based models [21]. This gap in implementation limits the ability to evaluate model efficacy in real-world situations, which in turn impedes the adoption of ML-based interventions for reducing adverse maternal outcomes at scale.

The goal of this review is to document evidence for the use of ML models for predicting the risk of maternal morbidity and mortality outcomes, and the translation of such models into applications in clinical use. The specific review objectives are to (1) describe ML models used to predict maternal morbidity and mortality outcomes from EMR data (RO1), (2) describe clinical applications that use ML models to predict the risk of severe maternal morbidity and mortality outcomes from EMR data in health care settings (RO2), and (3) describe factors influencing implementation of clinical applications that use ML models to predict the risk of severe maternal morbidity and mortality outcomes from EMR data in health care settings (RO3).

For the purpose of this review, we described EMRs as any data contained in health information systems used for clinical care. We included several types of EMR data, including administrative and billing data, patient demographics, social determinants of health, progress notes, vital signs, medical histories, diagnoses, obstetric history (eg, with timestamps of events like C-section), medications, immunization dates, allergies, radiology images, laboratory and test results, and other data (eg, neonatal and infant outcomes). Second, we described maternal morbidity and mortality as any adverse outcomes that occur during pregnancy and up to 1 year postpartum. We did not use specific maternal morbidity and mortality outcomes as inclusion criteria for the review. Rather, we categorized outcomes evaluated in the included studies post hoc. Third, we described ML models as computational approaches where patterns learned from historically collected data (ie, EMRs within the scope of this study) are used to make predictions on pregnancy-related outcomes using EMR data [22,23]. We were interested in:

1. Supervised models that use labeled data for learning patterns. Examples of models could include linear and logistic regression, support vector machines (SVMs), decision trees, ensemble methods (eg, random forests), k-nearest neighbor, naive Bayes classifiers, or other supervised learning models.
2. Unsupervised learning models that seek to identify natural relationships or groupings from unlabeled data. Examples of models could include clustering analysis, density estimation, dimensionality reduction, or other unsupervised learning models.
3. Semisupervised models that predict patterns from labeled and unlabeled data.
4. Representation (deep) learning models that engage in data-driven learning and use artificial neural networks.

We only included models that had been evaluated for metrics such as performance (accuracy, sensitivity, specificity, and area under the curve [AUC], misclassification rate, or another performance metric), fairness (demographic parity, equalized odds, or another fairness metric), interpretability (feature summary statistics and visualization, model specific or model agnostic interpretations, or others such as intrinsic or post hoc models).

Finally, we defined clinical applications as any software feature, program, application (app), or other digital tools (eg, clinical decision support tools and risk calculators) that presented the predictions from the ML models to a health provider or pregnant patient for the purpose of managing clinical care in actual practice in health care settings. Applications that were developed for but not implemented in health care settings for clinical care were excluded.

Several previous reviews have examined the use of ML models for predicting the risk of maternal morbidity and mortality outcomes; however, key gaps in evidence synthesis remain. Some previous reviews have assessed pregnancy outcomes and the identification of complications in pregnancy, but not the translation of risk prediction models into clinical applications in health care settings [24,25]. Other reviews assess predictions of a specific maternal outcome, for example, preterm birth, hypertension, postpartum hemorrhage, gestational diabetes mellitus, using different ML techniques, but not maternal morbidity and mortality broadly [26,27]. Other reviews document ML methods or the implementation of artificial intelligence and its evolution over the years in the field of maternal health, but do not focus on risk prediction [18,28,29]. A more recent review examined artificial intelligence–based clinical decision support tools, but the review was not limited to risk prediction, EMR data sources, or maternal outcomes [30]. To bridge those gaps in the literature, the objective of this review is to examine the full pipeline of studies from the development of ML models used to predict maternal morbidity and mortality outcomes from EMR data to factors affecting the implementation of applications using those models in clinical practice.

## Methods

The methods of this scoping review were adapted from the Joanna Briggs Institute’s methodology for scoping reviews [31]. The methods of the review are reported in accordance with the PRISMA-ScR (Preferred Reporting Items for Systematic Reviews and Meta-Analyses extension for Scoping Reviews) reporting checklist presented in Checklist 1.

### Eligibility Criteria

The eligibility criteria used to screen studies in this review are presented in Textbox 1.

Textbox 1. **Eligibility criteria for studies included in the review on implementation of machine learning (ML) models to predict maternal morbidity and mortality outcomes from electronic medical record (EMR) data.**

**Inclusion criteria for review objective 1 (RO1):**

- Described the use of ML models for predicting the risk of one or more maternal morbidity and mortality outcomes;
- Used EMR data as inputs in the ML models; and
- Were conducted using data from pregnant patients or their children in health care settings.

**Exclusion criteria for RO1:**

- Solely used non-EMR data (eg, administrative data from insurance companies) as inputs for the ML models.
- Only examined neonatal outcomes or other maternal features (eg, ultrasound images or histology or placental pathology images).

**Inclusion criteria for review objective 2 (RO2):**

- Met the criteria for RO1, and
- Described clinical applications that were implemented in health care settings,
- To improve clinical care or service delivery during pregnancy or to impact pregnant patients’ health or behavior;
-  irrespective of whether they were integrated with the EMR or stand-alone applications; and
-  irrespective of their modality of delivery (eg, tablet, mobile phone, computer, etc)

**Inclusion criteria for review objective 3 (RO3):**

- Met the criteria for RO2;
- Described implementation factors (eg, technological, behavioral, leadership and governance, and financial) that affected the adoption, scale-up, integration, or sustainability of clinical applications; and/or
- Described empirical evidence on factors influencing the implementation of clinical applications in health care settings.

**Exclusion criteria for RO3:**

- Only included nonempirical or anecdotal descriptions (eg, in the discussion section of publications) of implementation factors without associated empirical data.

**Inclusion criteria for participants:**

- Any pregnant patient from the time of their last menstrual period and up to 1 year postpartum or whose EMR data were used in the ML model, or who may benefit from a clinical application where such models are used.
- Any health care providers (including prenatal providers, advanced practice providers, anesthesiologists, critical care, family medicine, and emergency department providers) or staff: who work in health care settings, and who used clinical applications that leverage ML models to predict risk of maternal morbidity and mortality outcomes in the delivery of clinical care.

**Inclusion criteria for context:**

- This review was limited to studies from health care settings where EMRs were used. We described health care settings as hospitals or other outpatient settings that cared for pregnant patients and documented their care using EMRs.

**Exclusion criteria for context:**

- There were no exclusions based on geographic units (eg, country).

**Inclusion criteria for sources:**

- For the purposes of this review, we only included peer-reviewed original research articles from journals, which were anticipated to contain methodological details of interest in this review. We included quantitative studies irrespective of their study design. Qualitative studies were eligible for inclusion for RO3 if the goal of qualitative data collection was to generate empirical data on the factors that affect implementation of clinical applications. We included qualitative studies irrespective of the underpinning theoretical framework (eg, phenomenology and action research).

**Exclusion criteria for sources:**

- We excluded review articles, text and opinion papers, conference papers, and any non–peer-reviewed papers as they do not consistently describe individual study methods in detail.
- We excluded any studies that were noted to be retracted on the journal website.

### Search Strategy

A detailed search strategy was developed with assistance from a health sciences librarian and included terms related to maternal health, artificial intelligence, and EMRs. We searched the following databases for studies meeting the eligibility criteria for the review: PubMed, CINAHL plus with full text (EBSCOhost), Scopus, Embase, and IEEE Xplore. We conducted an initial limited search in PubMed to identify studies on the topic. We used the text words in the titles and abstracts of relevant articles and the index terms used to describe the studies to develop a full search strategy for the other databases (refer to Tables S1A-S1E in Multimedia Appendix 1 for the detailed list of search terms by database). The search strategy, including all identified keywords and index terms, was adapted for each included database or information source and run on February 20, 2023. We did not search any gray literature resources (eg, Google Scholar) or trial databases during the initial search. We only included studies published in English. Studies in other languages were eligible to be considered if a certified English translation was available from the publishing journal. For RO2, a second search was conducted where the final included studies from the initial search were entered into Google Scholar, and the list of citing studies was evaluated for eligible studies. This step was included in the event that model development and clinical applications were described in distinct follow-on manuscripts or published in different journals.

### Study Selection

Following the initial search, we collated and uploaded all identified citations into EndNote v.19 (Clarivate Analytics) and removed duplicates. Titles and abstracts of each study were screened in Covidence by 2 independent reviewers for assessment against the inclusion criteria for the review. Two independent reviewers assessed the full text of selected citations in detail against the inclusion criteria. Reasons for exclusion of studies during full-text screening were recorded. Any discordance in screening was resolved through discussion or with an additional independent reviewer. The search results and the study inclusion process are reported in a PRISMA-ScR flow diagram. The findings of the Google Scholar search were documented in a spreadsheet.

### Data Extraction

Data were extracted from each included study by 1 reviewer using a data extraction spreadsheet (Table S2 in Multimedia Appendix 1). The data extracted included details about the participants, concept, context, study methods, and key findings relevant to the review questions (eg, clinical features). In the initial stages of data extraction, reviewers piloted the data extraction spreadsheet. Reviewers met regularly to discuss and clarify any extracted content as needed.

### Data Analysis and Presentation

The data extracted from the study were summarized narratively and visually (in tables and figures) and described by review objectives. We described the geographic distribution of studies as well as the representativeness of health care settings (eg, academic medical centers, community clinics, etc) included in studies. Where data were available, we described participant type (eg, pregnant and postpartum) and characteristics (eg, insurance status, race, ethnicity, income status, clinical characteristics, etc). We described outcomes up to 1 year postpartum, types of ML methods and tools used, and software or programming languages used for ML tasks.

## Results

**Figure 1.**
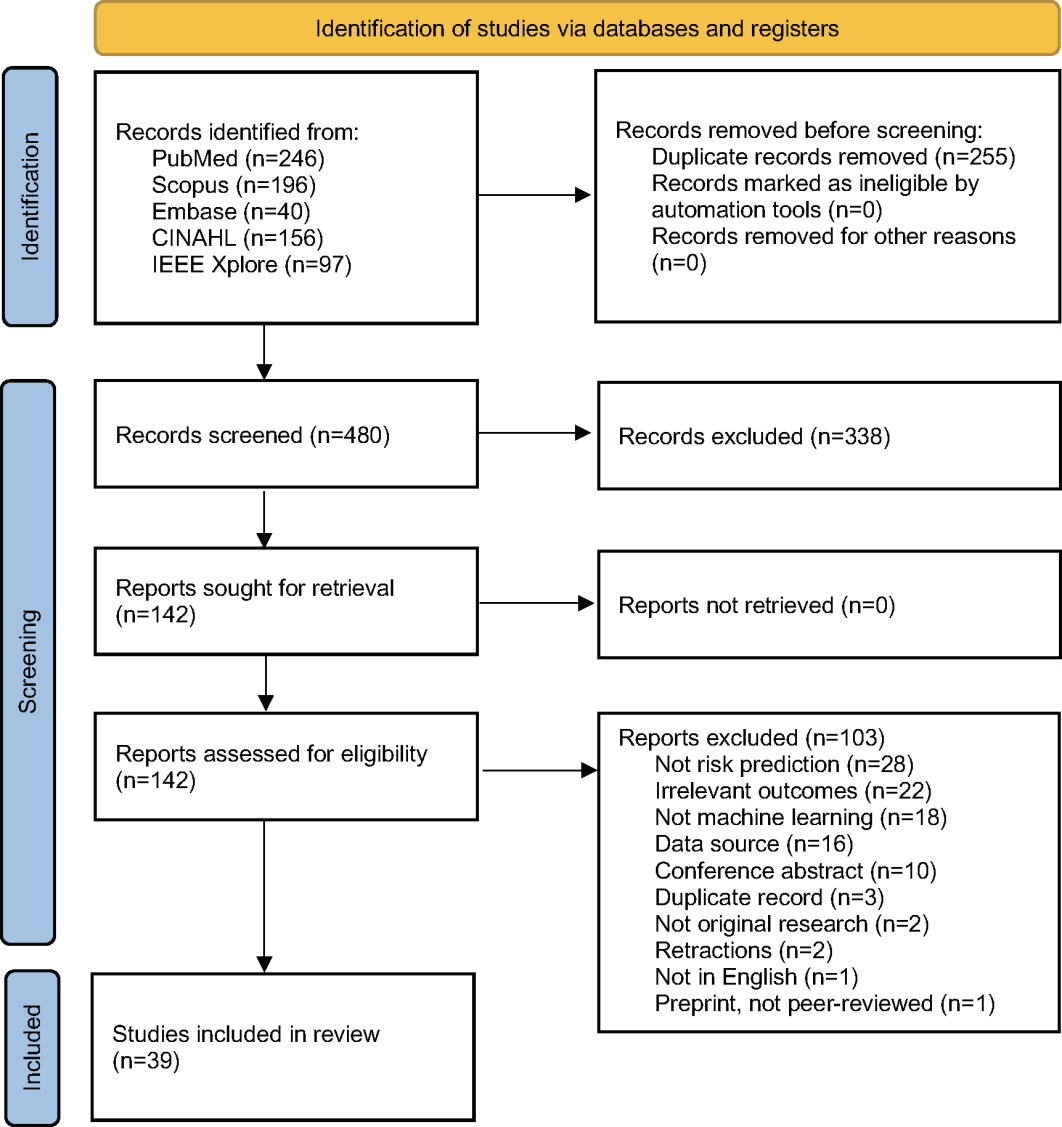
PRISMA (Preferred Reporting Items for Systematic Reviews and Meta-Analyses) flow diagram for scoping review on implementation of machine learning models to predict maternal morbidity and mortality outcomes from electronic medical record data [32]. This work is licensed under CC BY 4.0.

The PRISMA (Preferred Reporting Items for Systematic Reviews and Meta-Analyses) [32] flow diagram depicting steps in study selection is presented in Figure 1. The detailed database-oriented search strategy identified 480 deduplicated studies, which were screened for relevance using their titles and abstracts. After title and abstract screening, 142 studies were included for full text review. At the full-text review stage, 103 studies were excluded as they did not meet one or more eligibility criteria for the review (RO1). For instance, many excluded studies did not include risk prediction (28 studies), maternal and morbidity outcomes (22 studies), or ML approaches (18 studies). Finally, 39 studies met eligibility criteria and were included in the scoping review [27,33-70]. The Google Scholar search for citing studies was conducted from October 1‐12, 2024. A range of 0‐246 citing studies was identified, but none met the criteria for RO2 or RO3 (Table S3 in Multimedia Appendix 1).

Key characteristics of included studies are shown in Figure S1 in Multimedia Appendix 1 and details of individual studies are summarized in Table S4 in Multimedia Appendix 1. Most of the included studies (34 studies) were conducted in just 3 countries: United States (17 studies) [27,35,37-40,45,48,51,55,56,58,61,63,64,69,70], China (11 studies) [36,41,43,48,49,53,54,59,65,67,68], and Israel (6 studies) [34,42,44,46,52,62]. Over half of included studies were published in 2022 or later (22 studies) [27,35-38,41,46,48,50,51,53,54,56,58,60,61,63,64,66-68,70] alone compared with previous years (pre-2022, 17 studies) [33,34,39,40,42-45,47,49,52,55,57,59,62,65,69]. Most included studies used a cohort study design (33 studies) [27,33-37,39,40,42,44-54,56-58,60-66,68-70] as opposed to cross-sectional or case-control designs (6 studies) [37,41,43,55,59,67]. More included studies focused on identifying the risk of pregnancy outcomes and delivery outcomes (34 studies) [27,34-43,45-52,54-62,64-68,70] compared with postpartum outcomes (5 studies) [33,44,53,63,69]. The top 3 most common outcomes for risk prediction in included studies were cardiovascular risks and hypertensive disorders of pregnancy (9 studies) [36,43,45,47-50,54,61], gestational diabetes (7 studies) [34,46,51,57,59,65,66], and postpartum hemorrhage (6 studies) [40,41,53,56,63,70].

Studies varied in their approach for cohort selection. Criteria used to identify maternal status in EMR included the use of *ICD-9* (*International Classification of Diseases, Ninth Revision*) or *ICD-10* (*International Statistical Classification of Diseases, Tenth Revision*) codes. For example, Clapp 2022 [38] used *ICD-10* Z37 code to identify delivery encounters. In most cases, clinical criteria were used to select cohorts with specific outcomes of interest (eg, gestational diabetes mellitus and hypertension). Ages of pregnant patients in the cohorts varied by study. Some studies restricted their cohorts to pregnant patients who were aged 18 years or older, while others included pregnant patients as young as 12 years [48], reported specific age ranges (eg, 18‐45 years old [46]), or only reported mean age. Due to restrictions based on the eligibility criteria for this review, the maximum length of postpartum follow-up was 1 year postdelivery. In 7 studies [27,42,46,52,54,58,68], the cohort of pregnant patients was limited to those with singleton pregnancies, but others included twins and higher-order multiple pregnancies.

Key details of data types and features in individual studies are summarized in Tables S5 and S6 in Multimedia Appendix 1. In most included studies, the EMR platform referenced a hospital-specific system (32 studies) [27,33-36,38,41-45,47-54,56,58-60,62-67,69,70]. In addition to routine clinical data, data types included medical images (eg, ultrasounds and 8 studies) [35,42,50,53,54,60,62,67], biological markers (4 studies) [27,44,57,65], data on social determinants of health (12 studies) [27,35,37,39,44,51,58,61,64,66,69,70] or other data (eg, billing codes, unstructured data, and 8 studies) [27,44,50,51,57,61,62,69]. More studies considered greater than 25 features (29 studies) [27,33-42,44,45,47-53,56,57,59,63-65,67,69,70] as inputs for the final model as opposed to 25 or fewer features (10 studies) [43,46,54,55,58,60-62,66,68]. The number of features included in the final model ranged from 7 features [47,65] to 176 features [51].

Summaries of the use of feature selection and feature construction methods are shown in Figure S2 in Multimedia Appendix 1, and details of individual studies are shown in Table S7 in Multimedia Appendix 1. The number of records in the included studies ranged from 400 to 588,622. Among included studies, 22 studies reported the use of feature selection [33-36,39-41,43,45,47-51,58-60,63,65,67,69,70], while 28 studies reported the use of feature construction [27,33-41,45,47-49,51,52,54,56,58-62,64-66,69,70]. Among the 22 studies reporting the use of feature selection, 2 studies [33,34] did not describe the methods used. Among the methods described for feature selection, the top category was automated methods (13 studies) [36,39,41,43,45,47,48,58-60,65,67,70]. Among the 28 studies reporting use of feature construction, 22 studies reported the use of simple feature construction [33,35,36,38,39,41,45,47-49,51,52,54,58-62,64-66,69], 3 studies reported the use of complex feature construction [27,37,56], and 3 studies did not report the type of feature construction used [34,40,70]. The top categories of individual feature construction methods were standardization (5 studies) [35,49,54,61,62] and normalization (4 studies) [59,61,69,70], and studies often used multiple methods.

A summary of ML models used for risk prediction is presented in Figure S3 in Multimedia Appendix 1 and details of individual studies are shown in Table S8 in Multimedia Appendix 1. Data were labeled by computable phenotypes in three-fourths of the included studies (30 studies) [27,33-37,39,40,43-49,53,54,56,58-60,62-70]. Most studies tested multiple ML methods to train the final risk prediction model, but top methods tested included boosting methods (24 studies) [27,33,34,40-42,44-49,51-53,56,58,63,64,66-70], logistic regression (20 studies) [27,36,39-41,43,45-47,49,51,53,54,56,59,60,63,65,67,69] and random forest (15 studies) [36,40,41,47,49,51,53,54,56,58,63,64,66,67,69]. Final ML models with best performance included boosting methods (18 studies) [27,33,34,42,44-49,51-53,56,63,66,67,70], random forest (7 studies) [36,41,51,54,56,58,64] and logistic regression (5 studies) [40,53,60,65,69]. ML models were evaluated using cross-validation (23 studies) [33,35,36,38,39,42,45,48,49,51-54,56,59,60,62,64,65,67-70], training and testing sets (19 studies) [27,35,37,39-41,43,44,46,50,55,58,62,63,65-68,70], and validation sets (16 studies) [27,33,34,37,39,40,44,45,47-49,51,62,63,66,69]. The most common metrics used to assess model performance included AUC or area under the precision-recall curve (AUPRC; 33 studies) [27,33-42,44-49,51-56,59,60,62-65,67-70], sensitivity, recall, or false positive rate (25 studies) [33,35,36,38,40-42,44-50,53-55,57,59,60,63,67-70], true negative rate, specificity, or false negative rate (18 studies) [33,35,36,40-42,44,46-49,53,55,60,67-70], positive predictive value, precision, or negative predictive value (15 studies) [35,38,40-42,44-46,48-50,54,57,69,70] and accuracy (15 studies) [35,43,46,47,49,50,54,55,57-59,63,66-68]. CIs or significance results on performance metrics were calculated in 25 studies [33,34,36-39,41,42,44,46,51-54,56,58-60,62,63,65-67,69,70]. Software tools used in included studies were Python (18 studies) [27,42-46,48-50,52-54,58,66-70], R (R Foundation for Statistical Computing; 15 studies) [35-38,41,43,44,47-49,56,58,59,62,63], and SPSS (IBM Corp; 8 studies) [41-43,50,52,54,64,67]; many studies used more than 1 software tool.

**Figure 2.**
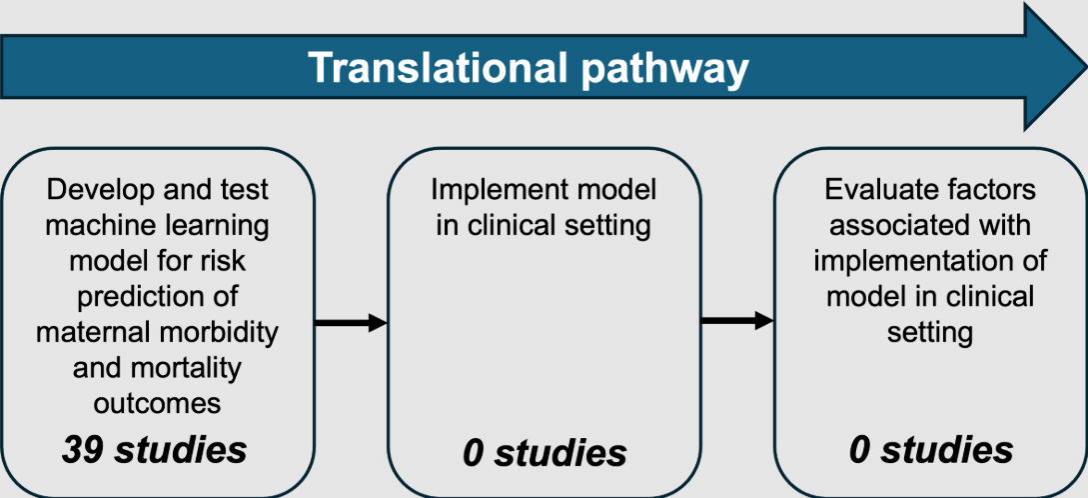
Studies identified in the translation pathway from model development to real-world implementation in a scoping review of machine learning models to predict maternal morbidity and mortality outcomes from electronic medical record data.

Figure 2 summarizes the translational pathway from developing risk prediction models to real-world clinical applications. The included studies did not present clinical applications of ML models (RO2). Artzi et al [34] described the development of a 9-item screening tool to predict the risk of gestational diabetes mellitus during pregnancy. However, only a retrospective validation of the screening tool was presented, and the tool was not implemented or evaluated in a clinical setting. A citation search of the 39 included studies yielded no further publications relevant to clinical applications of models (RO2) or associated implementation factors (RO3).

## Discussion

### Principal Findings

Our scoping review examined the use of ML models for predicting the risk of maternal morbidity and mortality outcomes, and the translation of such models into applications in clinical use. Related to RO1, we identified 39 studies that developed and tested ML models for predicting the risk of maternal morbidity and mortality outcomes using EMR data. We found few studies of postpartum maternal morbidity or mortality outcomes, and most studies were conducted in China, Israel, or the United States. A number of included studies only considered pregnancies that resulted in a live birth during cohort selection, introducing a potential bias in modeling results [71,72]. Included studies reported a variety of models for risk predictions, with LASSO (Least Absolute Shrinkage and Selection Operator), boosting methods, and random forest being reported as the best-performing methods. Related to ROs 2 and 3, our review highlighted significant gaps in the translational pipeline; while all included studies used ML to predict maternal morbidity and mortality outcomes, no studies used these models in a clinical application that was deployed in practice.

Our review findings highlight several gaps in research. Despite increasing recognition of the high and disparate rates of maternal mortality, no studies included in our review examined maternal mortality directly, and only 1 study (Chen et al [36]) included mortality as part of a composite outcome [73]. In addition, the majority of studies focused on the pregnancy and delivery period, while few studies examined postpartum outcomes occurring outside the delivery hospitalization, highlighting an area for future work. Recent work has demonstrated that the majority of pregnancy-related deaths occur after delivery, and there are many disparities in postpartum care [10,74]. We hypothesize that the focus on antenatal and delivery outcomes may be due to data availability. While it is relatively easy to identify delivery hospitalizations at a given center, postpartum patients may receive care across multiple providers and centers, complicating data aggregation and outcome ascertainment. Among studies that used maternal age as a criterion for cohort selection, we identified inconsistencies in age ranges used for cohort selection or insufficient reporting of details for reproducibility in other studies. Data analysis programming code for the final models was not readily cited in the publications, limiting reproducibility of the research. Inadequate and inconsistent reporting was also observed in the area of feature engineering, where the methods used were not explained or named by all studies. Many studies did not implement external validation strategies, techniques, or sensitivity analyses. Such limitations raise concerns about the statistical rigor or robustness of the results published in the included studies.

Future efforts should focus on less commonly studied maternal morbidity and mortality outcomes, including those in the postpartum period. One mechanism to encourage research during the postpartum period may be through specific funding opportunities prioritizing the development of ML models in that timeframe. As noted above, a key challenge in developing such models will be the availability of data linking pregnancy and postpartum visits, and potentially linking across varied providers and health care settings. Specific efforts may be necessary to build integrated data warehouses that securely triangulate data from multiple sources. Doing so will also be important from the perspective of increasing geographic representation in studies and ensuring that the predictive models developed are generalizable in wider contexts. Achieving generalizability will require attention to data provenance, security, and integrity, as well as establishment of legal, regulatory, and interoperability frameworks that facilitate rapid collation and analysis of data.

Future efforts should also consider mechanisms to increase transparency and reproducibility of studies through consistent and detailed reporting of methodology. Only 6 of the 39 studies in our review described the use of a reporting guideline, with all using Transparent Reporting of a multivariable prediction model for Individual Prognosis Or Diagnosis (TRIPOD) guideline [38,39,49,58,64,70,75]. Peer-reviewed journals and conferences often recommend the use of study design-specific reporting guidelines, and the use of such reporting guidelines has been shown to increase transparency and quality of reporting of methods and findings. Several reporting guidelines are available to describe the use of artificial intelligence approaches, including ML, for clinical outcome prediction and modeling. These include TRIPOD-AI (Transparent Reporting of a multivariable prediction model for Individual Prognosis Or Diagnosis-Artificial Intelligence) [76], CONSORT-AI (Consolidated Standards of Reporting Trials-Artificial Intelligence) [77], SPIRIT-AI (Standard Protocol Items: Recommendations for Interventional Trials-Artificial Intelligence) [78], and MINIMAR (Minimum Information for Medical AI Reporting) [79] among others. The Enhancing the Quality and Transparency of Health Research (EQUATOR) network is a global initiative that provides comprehensive and up-to-date information on currently available, validated reporting guidelines [80].

Our findings highlight a need to expand efforts to translate, implement, and evaluate the use of the ML models in clinical practice. Although several published papers [81,82] suggested a translational path to successfully incorporate ML innovations into clinical care, we found no publications describing implementation of maternal morbidity and mortality ML models in clinical care. One proposed pathway approach may include a phased strategy that includes planning, design, testing and implementation, evaluation, and sustainability. In particular, the evaluation phase should focus on enhancing features and functionalities based on clinicians’ requests, gradual roll-out of the tool both horizontally and vertically by increasing the number of clinicians and the amount and diversity of training data, implementing performance and safety audits to address data quality and algorithmic biases [83], ensuring system safety from any adversarial effects, and undertaking formative or summative evaluation as necessary for sustainability includes enacting a stewardship, governance, and regulatory framework, securing financial investments, incorporating technological improvements, ensuring continuous capacity building activities for clinicians, and performing periodic knowledge dissemination. In addition to the above implementation framework, other relevant frameworks like the TRIPOD-AI statement for predictive modeling or HL7 (Health Level Seven) FHIR (Fast Healthcare Interoperability Resources) standards for data interoperability could support the structured design, development, and deployment of ML models in real-world settings. It is essential to remember that a human-centered design approach is critical to the success of such tools. Adherence to stewardship, governance, and regulatory frameworks would be required in this workflow for successful translation of ML innovations into clinical practice.

Study limitations are the inclusion of only peer-reviewed journal articles and those published in English, which may have led to the exclusion of novel research findings presented in conference settings or in other languages. While we tried to be comprehensive with the selection of search terms and databases, we may have missed studies published elsewhere or using alternative terms. Finally, our findings should be interpreted with caution as we did not formally evaluate the risk of bias in the included studies. Review strengths are the inclusion of all maternal morbidity and mortality outcomes, lack of geographic exclusions, and examination of the full translational pipeline of studies from the model’s development to application in clinical practice.

### Conclusions

In summary, our scoping review identified 39 studies that developed and tested ML models for predicting the risk of maternal morbidity and mortality outcomes using EMR data. However, we found significant gaps in the translation of the models to create a clinical application that was deployed in practice. Future studies should focus on evaluating the implementation of ML models in clinical settings as part of its translational pathway for improving patient outcomes. While the growth in efforts to use ML models for the prevention of maternal morbidity and mortality are encouraging, increasing the representativeness of maternal morbidity and mortality outcomes and country settings of studies is essential for understanding the scalability of ML approaches, including in low-resource settings where the burden of maternal mortality is high.

## Supplementary material

10.2196/68225 Multimedia Appendix 1 Additional information.

10.2196/68225 Checklist 1 PRISMA-ScR (Preferred Reporting Items for Systematic Reviews and Meta-Analyses extension for Scoping Reviews) checklist.

## Abbreviations

AUC: area under the curve
AUPRC: area under the precision-recall curve
CONSORT-AI: Consolidated Standards of Reporting Trials - Artificial Intelligence
EMR: electronic medical record
EQUATOR: Enhancing the Quality and Transparency of Health Research
FHIR: Fast Healthcare Interoperability Resources
HL7: Health Level Seven
*ICD-10*: *International Statistical Classification of Diseases, Tenth Revision*
*ICD-9*: *International Classification of Diseases, Ninth Revision*
LASSO: Least Absolute Shrinkage and Selection Operator
MINIMAR: Minimum Information for Medical AI Reporting
ML: machine learning
PRISMA: Preferred Reporting Items for Systematic Reviews and Meta-Analyses
PRISMA-ScR: Preferred Reporting Items for Systematic Reviews and Meta-analyses extension for scoping review
RO: review objective
SPIRIT-AI: Standard Protocol Items: Recommendations for Interventional Trials - Artificial Intelligence
SVM: support vector machine
TRIPOD: Transparent Reporting of a multivariable prediction model for Individual Prognosis Or Diagnosis
TRIPOD-AI: Transparent Reporting of a multivariable prediction model for Individual Prognosis Or Diagnosis - Artificial Intelligence
