# Supplementary material for: Machine Learning Models to Predict Risk of Maternal Morbidity and Mortality From Electronic Medical Record Data: Scoping Review
Source: J Med Internet Res. 2025 Aug 14;27:e68225. doi: 10.2196/68225 (PMC12352520; doi:10.2196/68225)
Supplement: Multimedia Appendix 1 [file jmir-v27-e68225-s001.docx]

**SUPPLEMENTARY FILES**

**SUPPLEMENTARY FIGURE 1: Characteristics of studies included in the scoping review (n=39) on implementation of machine learning models to predict maternal morbidity and mortality outcomes from electronic medical record data.**

**
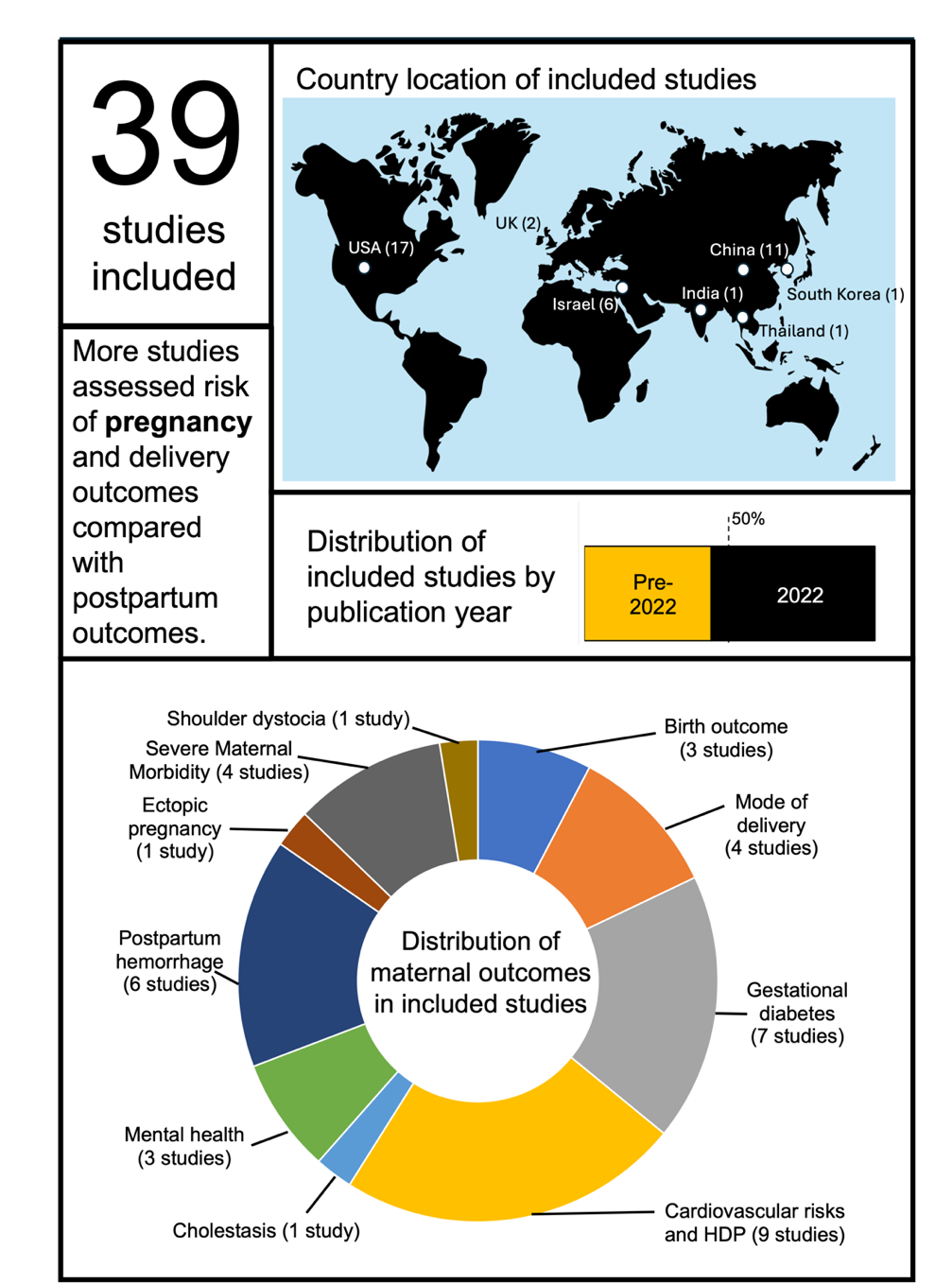
**

**Abbreviations:** HDP: Hypertensive Disorders of Pregnancy

**SUPPLEMENTARY FIGURE 2: Feature engineering in studies included in the scoping review (n=39) on implementation of machine learning models to predict maternal morbidity and mortality outcomes from electronic medical record data.**

**
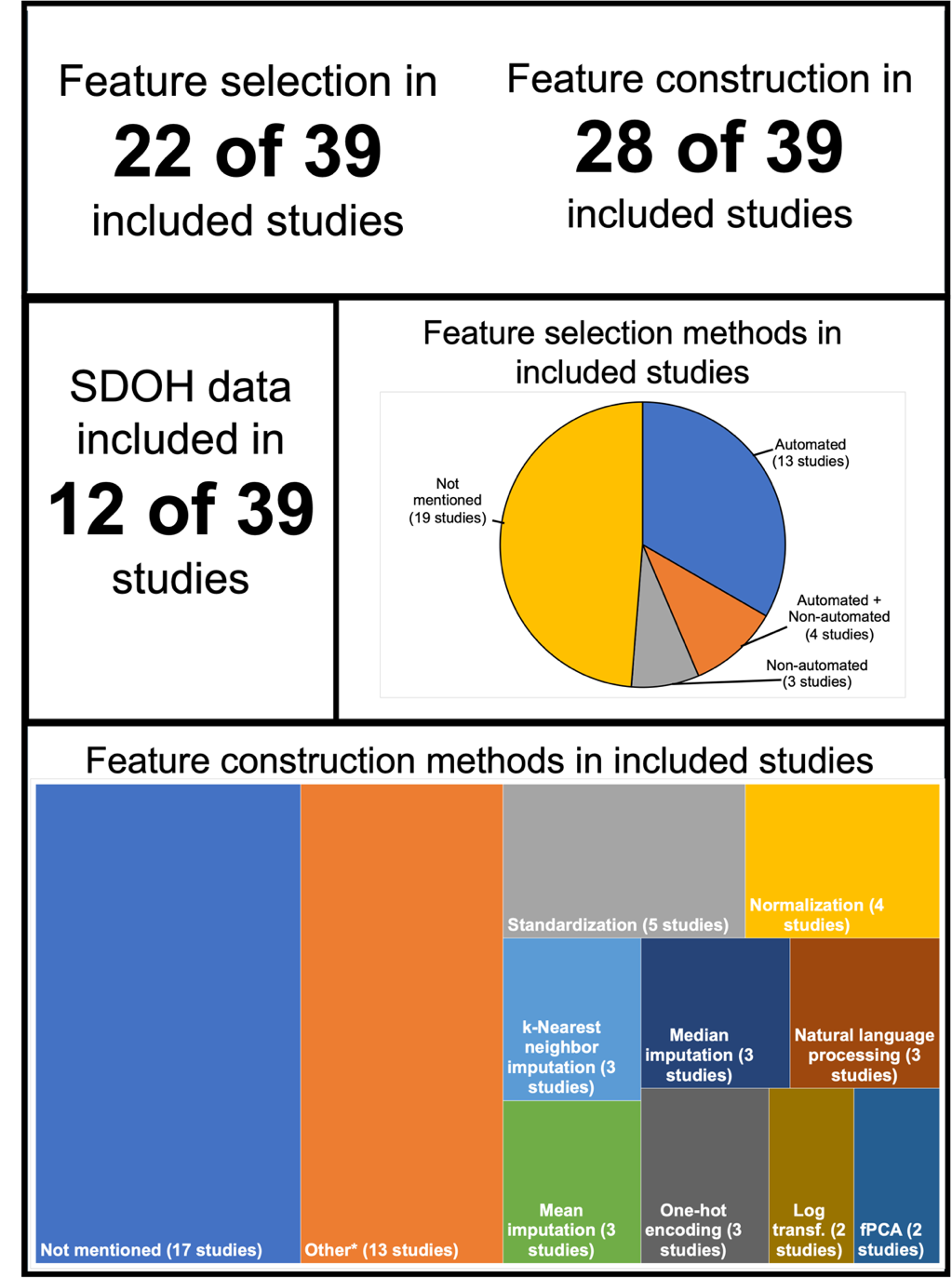
**

**Abbreviations:** fPCA: Functional Principal Component Analysis.

**Footnotes:** *Other includes box-cox transformation, clustering, propensity score matching, text mining, random forest imputation etc.

Some included studies used multiple methods, so the total exceeds 39.

**SUPPLEMENTARY FIGURE 3: Machine Learning methods used in studies included in the scoping review (n=39) on implementation of machine learning models to predict maternal morbidity and mortality outcomes from electronic medical record data.**

**
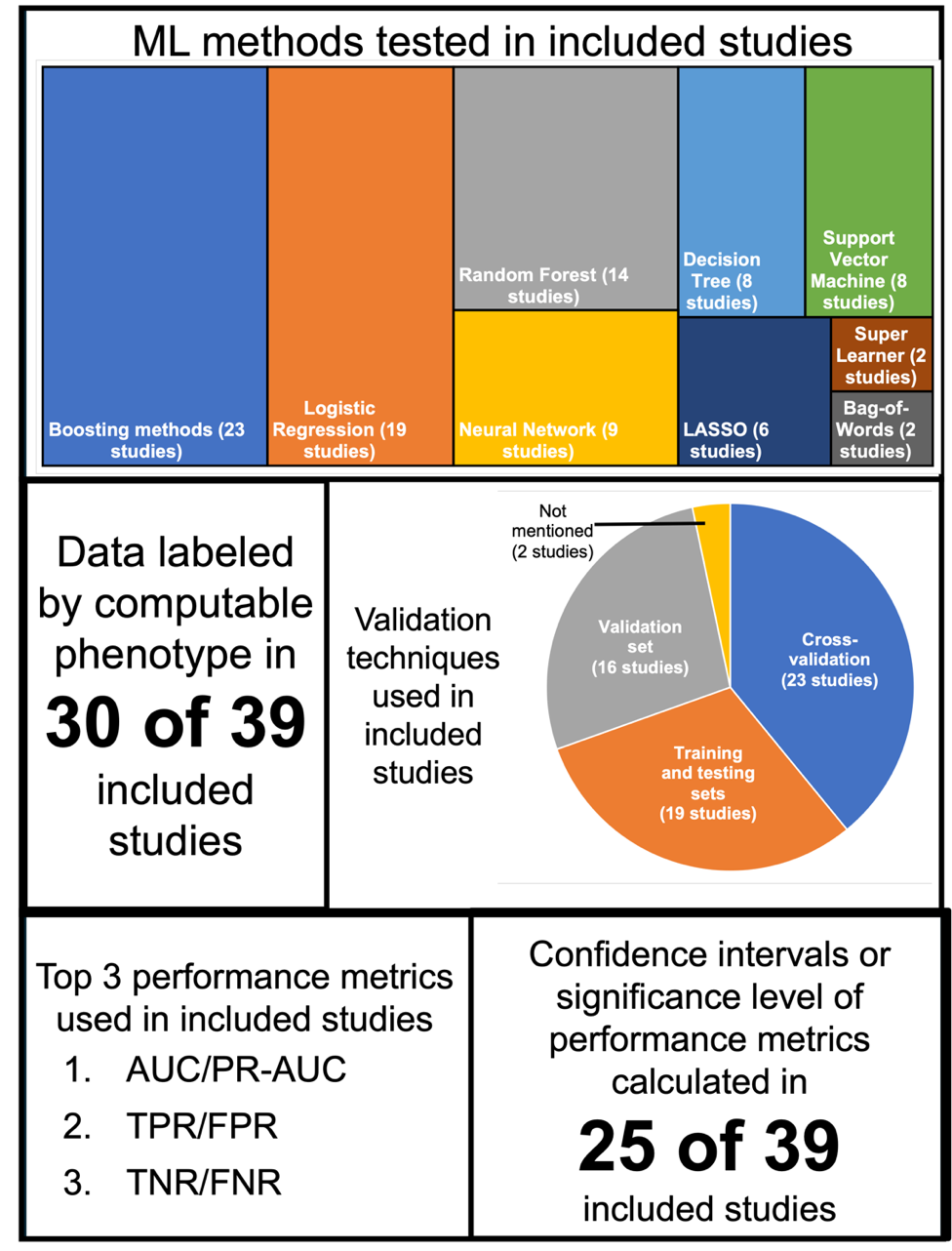
**

**Abbreviations:** ML: Machine Learning; AUC: Area Under the Curve; PR-AUC: area under the precision recall curve; TPR: True Positive Rate; FPR: False Positive Rate; TNR: True Negative Rate; FNR: False Negative Rate

**Footnotes:** Some included studies used multiple methods, so the total exceeds 39.

**SUPPLEMENTARY TABLE 1: Search strategy report for scoping review on implementation of machine learning models to predict maternal morbidity and mortality outcomes from electronic medical record data.**

Date: 2/20/2023

**SUPPLEMENTARY TABLE 1A: PubMed**

| Set# |  | Results |
| --- | --- | --- |
| 1 | "Maternal Health"[Mesh] OR "Obstetrics"[Mesh] OR "Prenatal Care"[Mesh] OR "Pregnancy"[Mesh] OR "Pregnant Women"[Mesh] OR "Postpartum Period"[Mesh] OR "Mothers"[Mesh] OR "Maternal Welfare"[Mesh] OR "Maternal Health Services"[Mesh] OR mother[tiab] OR mothers[tiab] OR maternal[tiab] OR obstetric[tiab] OR obstetrics[tiab] OR prenatal[tiab] OR perinatal[tiab] OR postnatal[tiab] OR peripartum[tiab] OR postpartum[tiab] OR puerperium[tiab] OR pregnant[tiab] OR pregnancy[tiab] OR pregnancies[tiab] OR "first trimester"[tiab] OR "second trimester"[tiab] OR "third trimester"[tiab] OR “fourth trimester”[tiab] |  |
| 2 | "Artificial Intelligence"[Mesh] OR "Data Mining"[Mesh] OR "Decision Making, Computer-Assisted"[Mesh] OR "Decision Support Systems, Clinical"[Mesh] OR "Pattern Recognition, Automated"[Mesh] OR “artificial intelligence”[tiab] OR “computational intelligence”[tiab] OR “machine intelligence”[tiab] OR “computer reasoning”[tiab] OR AI[tiab] OR “machine learning”[tiab] OR “deep learning”[tiab] OR “hierarchical learning”[tiab] OR “supervised learning”[tiab:~3] OR “semisupervised learning”[tiab:~3] OR “unsupervised learning”[tiab:~3] OR “natural language processing”[tiab] OR “training data”[tiab] OR “reinforcement learning”[tiab] OR “neural networks”[tiab] OR “neural network”[tiab] OR “data mining”[tiab] OR “text mining”[tiab] OR “computer-assisted decision”[tiab] OR “computer-assisted medical decision”[tiab] OR “computer-assisted diagnosis”[tiab] OR “computer-assisted diagnoses”[tiab] OR “computer-assisted therapy”[tiab] OR “computer-assisted therapies”[tiab] OR “risk calculator”[tiab] OR “risk calculators”[tiab] OR “clinical decision support”[tiab] OR “automated pattern recognition”[tiab] OR “pattern recognition system”[tiab] OR “pattern recognition systems”[tiab] |  |
| 3 | "Electronic Health Records"[Mesh] OR “health records”[tiab] OR “health record”[tiab] OR “medical records”[tiab] OR “medical record”[tiab] OR EHR[tiab] OR EMR[tiab] OR “patient record”[tiab] OR “patient records”[tiab] OR “hospital record”[tiab] OR “hospital records”[tiab] |  |
| 4 | #1 AND #2 AND #3 | 246 |

**SUPPLEMENTARY TABLE 1B: Scopus**

| Set# |  | Results |
| --- | --- | --- |
| 1 | TITLE-ABS (mother OR mothers OR maternal OR obstetric OR obstetrics OR prenatal OR perinatal OR postnatal OR peripartum OR postpartum OR puerperium OR pregnant OR pregnancy OR pregnancies OR "first trimester" OR "second trimester" OR "third trimester" OR “fourth trimester”) |  |
| 2 | TITLE-ABS (“artificial intelligence” OR “computational intelligence” OR “machine intelligence” OR “computer reasoning” OR AI OR “machine learning” OR “deep learning” OR “hierarchical learning” OR (supervised W/3 learning) OR (semisupervised W/3 learning) OR (unsupervised W/3 learning) OR “natural language processing” OR “training data” OR “reinforcement learning” OR “neural networks” OR “neural network” OR “data mining” OR “text mining” OR “computer-assisted decision” OR “computer-assisted medical decision” OR “computer-assisted diagnosis” OR “computer-assisted diagnoses” OR “computer-assisted therapy” OR “computer-assisted therapies” OR “risk calculator” OR “risk calculators” OR “clinical decision support” OR “automated pattern recognition” OR “pattern recognition system” OR “pattern recognition systems”) |  |
| 3 | TITLE-ABS (“health records” OR “health record” OR “medical records” OR “medical record” OR EHR OR EMR OR “patient record” OR “patient records” OR “hospital record” OR “hospital records”) |  |
| 4 | #1 AND #2 AND #3 | 196 |

**SUPPLEMENTARY TABLE 1C: Embase**

| Set# |  | Results |
| --- | --- | --- |
| 1 | 'maternal welfare'/exp OR 'obstetrics'/exp OR 'prenatal care'/exp OR 'pregnancy'/exp OR 'pregnant woman'/exp OR 'puerperium'/exp OR 'mother'/exp OR 'maternal health service'/exp OR mother:ti,ab,kw OR mothers:ti,ab,kw OR maternal:ti,ab,kw OR obstetric:ti,ab,kw OR obstetrics:ti,ab,kw OR prenatal:ti,ab,kw OR perinatal:ti,ab,kw OR postnatal:ti,ab,kw OR peripartum:ti,ab,kw OR postpartum:ti,ab,kw OR puerperium:ti,ab,kw OR pregnant:ti,ab,kw OR pregnancy:ti,ab,kw OR pregnancies:ti,ab,kw OR ‘first trimester’:ti,ab,kw OR ‘second trimester’:ti,ab,kw OR ‘third trimester’:ti,ab,kw OR ‘fourth trimester’:ti,ab,kw |  |
| 2 | 'artificial intelligence'/exp OR 'data mining'/exp OR 'decision support system'/exp OR 'automated pattern recognition'/exp OR ‘artificial intelligence’:ti,ab,kw OR ‘computational intelligence’:ti,ab,kw OR ‘machine intelligence’:ti,ab,kw OR ‘computer reasoning’:ti,ab,kw OR AI:ti,ab,kw OR ‘machine learning’:ti,ab,kw OR ‘deep learning’:ti,ab,kw OR ‘hierarchical learning’:ti,ab,kw OR supervised NEAR/3 learning OR semisupervised NEAR/3 learning OR unsupervised NEAR/3 learning OR ‘natural language processing’:ti,ab,kw OR ‘training data’:ti,ab,kw OR ‘reinforcement learning’:ti,ab,kw OR ‘neural networks’:ti,ab,kw OR ‘neural network’:ti,ab,kw OR ‘data mining’:ti,ab,kw OR ‘text mining’:ti,ab,kw OR ‘computer-assisted decision’:ti,ab,kw OR ‘computer-assisted medical decision’:ti,ab,kw OR ‘computer-assisted diagnosis’:ti,ab,kw OR ‘computer-assisted diagnoses’:ti,ab,kw OR ‘computer-assisted therapy’:ti,ab,kw OR ‘computer-assisted therapies’:ti,ab,kw OR ‘risk calculator’:ti,ab,kw OR ‘risk calculators’:ti,ab,kw OR ‘clinical decision support’:ti,ab,kw OR ‘automated pattern recognition’:ti,ab,kw OR ‘pattern recognition system’:ti,ab,kw OR ‘pattern recognition systems’:ti,ab,kw |  |
| 3 | 'electronic health record'/exp OR ‘health records’:ti,ab,kw OR ‘health record’:ti,ab,kw OR ‘medical records’:ti,ab,kw OR ‘medical record’:ti,ab,kw OR EHR:ti,ab,kw OR EMR:ti,ab,kw OR ‘patient record’:ti,ab,kw OR ‘patient records’:ti,ab,kw OR ‘hospital record’:ti,ab,kw OR ‘hospital records’:ti,ab,kw |  |
| 4 | #1 AND #2 AND #3 | 376 |
| 5 | #4 AND [embase]/lim NOT ([embase]/lim AND [medline]/lim) | 179 |
| 6 | #5 AND ('article'/it OR 'review'/it) | 40 |

**SUPPLEMENTARY TABLE 1D: CINAHL Plus with Full Text (EBSCO*host*)**

| Set# |  | Results |
| --- | --- | --- |
| 1 | MH "Maternal-Child Health" OR MH "Obstetrics" OR MH "Prenatal Care" OR MH "Pregnancy+" OR MH "Expectant Mothers" OR MH "Postnatal Period+" OR MH "Mothers" OR MH "Maternal Welfare" OR MH "Maternal Health Services+" OR mother OR mothers OR maternal OR obstetric OR obstetrics OR prenatal OR perinatal OR postnatal OR peripartum OR postpartum OR puerperium OR pregnant OR pregnancy OR pregnancies OR "first trimester" OR "second trimester" OR "third trimester" OR “fourth trimester” |  |
| 2 | MH "Artificial Intelligence+" OR MH "Data Mining+" OR MH "Decision Making, Computer Assisted+" OR MH "Decision Support Systems, Clinical" OR “artificial intelligence” OR “computational intelligence” OR “machine intelligence” OR “computer reasoning” OR AI OR “machine learning” OR “deep learning” OR “hierarchical learning” OR supervised N3 learning OR semisupervised N3 learning OR unsupervised N3 learning OR “natural language processing” OR “training data” OR “reinforcement learning” OR “neural networks” OR “neural network” OR “data mining” OR “text mining” OR “computer-assisted decision” OR “computer-assisted medical decision” OR “computer-assisted diagnosis” OR “computer-assisted diagnoses” OR “computer-assisted therapy” OR “computer-assisted therapies” OR “risk calculator” OR “risk calculators” OR “clinical decision support” OR “automated pattern recognition” OR “pattern recognition system” OR “pattern recognition systems” |  |
| 3 | MH "Electronic Health Records+" OR “health records” OR “health record” OR “medical records” OR “medical record” OR EHR OR EMR OR “patient record” OR “patient records” OR “hospital record” OR “hospital records” |  |
| 4 | #1 AND #2 AND #3 | 156 |

**SUPPLEMENTARY TABLE 1E: IEEE *Xplore***

| Set# |  | Results |
| --- | --- | --- |
| 1 | Mother* OR maternal OR obstetric* OR prenatal OR perinatal OR postnatal OR peripartum OR postpartum OR puerperium OR pregnan* OR trimester |  |
| 2 | AI OR intelligence OR “computer reasoning” OR “machine learning” OR “deep learning” OR “hierarchical learning” OR supervised NEAR/3 learning OR semi-supervised NEAR/3 learning OR unsupervised NEAR/3 learning OR “language processing” OR “neural network*” OR mining OR “computer-assisted” OR calculator* OR “decision support” OR pattern |  |
| 3 | records OR record OR EHR OR EMR |  |
| 4 | #1 AND #2 AND #3 | 425 |
| 5 | #4 AND Filters Applied: Journals, Early Access Articles | 97 |

**SUPPLEMENTARY TABLE 2: Data extraction fields for scoping review on implementation of machine learning models to predict maternal morbidity and mortality outcomes from electronic medical record data.**

| **Study identifiers** |
| --- |
| - Extracted by (reviewer initials) - Covidence # - Title - Study ID - Year published |
| **Study characteristics** |
| - Years study conducted - Place of study - Country of study - Study design |
| **Clinical characteristics** |
| - Population - Antepartum (yes/no) - Trimester (if available) - Intrapartum (yes/no) - Postpartum (yes/no) - Specific pregnancy/postpartum, neonatal outcome for which risk is predicted - Is age used as an eligibility-criteria for constructing the cohort? (Yes/No) - Age of population (specify if IQR or full range) - Other demographic or clinical characteristics used to generate cohort (i.e., used as eligibility criteria) |
| **Data characteristics** |
| - EMR data used? (Yes/No) - Name of EMR system - Medical images (recordings, echotomography, ultrasounds, resonance, etc.) included? (Yes/No) - Specify medical image elements - Biological markers included? (Yes/No) - Specify biological markers - SDOH data included? (Yes/No) (see (yellow triangle) comment for examples of SDOH data) - Specify SDOH data - Other data sources included?(Yes/No) - Specify other data sources - Specify other data elements |
| **Record characteristics** |
| - Description of records - Total number of records used for feature engineering (enter number only) - Total number of records used for predictive modeling (enter number only) - Features considered (write individually if <25, If >25, note class of features) - Features included in final model (write individually if <25, If >25, note class of features) - Number of features considered - Number of features included in final model |
| **Feature engineering** |
| - Whether feature selection done or not (Yes/No) - Feature Selection methods used (e.g., Rankers, Wrappers, other; Rankers includes Correlation, Mutual Information, others) - Whether feature construction done or not (Yes/No) - Type of feature construction/engineering (Simple/Complex) - Simple feature construction methods used (log, normalization, PCA, SVD...) - Complex feature construction methods used (includes Embeddings by DNN, as well as Clustering, Latent Dirichlet Allocation, other latent variable methods, and Predicate Invention) - Comments |
| **Machine learning methods** |
| - ML methods tested for prediction model - ML methods finally selected (i.e., one with best performance) - Were data labeled by computable phenotype (Yes/No) - Validation techniques used - Performance metrics used - Were confidence intervals or significance results on performance metrics calculated? (Yes/No) - Software/tools used for the machine learning tasks |
| **RO2** |
| - Clinical applications using ML implemented? Yes/No - Details of clinical application |
| **RO3** |
| - Implementation factors examined? Yes/No |
| **Notes** |
| - Please add any notes or general impressions that may help with discussion section or add more context to data extracted for papers |

**SUPPLEMENTARY TABLE 3: Results of google scholar citation search for studies included (n=39) in the scoping review on implementation of machine learning models to predict maternal morbidity and mortality outcomes from electronic medical record data.**

| **Author Year** | **Title** | **Date of google scholar search** | **Number of papers that cite this paper** | **Citing paper implements model in clinical practice? (RO2)** | **Citing paper describes implementation factors? (RO3)** |
| --- | --- | --- | --- | --- | --- |
| Abraham 2022 | Dense phenotyping from electronic health records enables machine learning-based prediction of preterm birth | Oct 3, 2024 | 23 | No | No |
| Amit 2021 | Estimation of postpartum depression risk from electronic health records using machine learning | Oct 12, 2024 | 36 | No | No |
| Artzi 2020 | Prediction of gestational diabetes based on nationwide electronic health records | Oct 12, 2024 | 246 | No | No |
| Cartus 2022 | Can Ensemble Machine Learning Improve the Accuracy of Severe Maternal Morbidity Screening in a Perinatal Database? | Oct 4, 2024 | 6 | No | No |
| Chen 2022 | Prediction of Adverse Outcomes in De Novo Hypertensive Disorders of Pregnancy: Development and Validation of Maternal and Neonatal Prognostic Models | Oct 12, 2024 | 6 | No | No |
| Clapp 2021 | Derivation and external validation of risk stratification models for severe maternal morbidity using prenatal encounter diagnosis codes | Oct 4, 2024 | 5 | No | No |
| Clapp 2022a | Natural language processing of admission notes to predict severe maternal morbidity during the delivery encounter | Oct 4, 2024 | 13 | No | No |
| Clapp 2022b | Comparison of Natural Language Processing of Clinical Notes with a Validated Risk-Stratification Tool to Predict Severe Maternal Morbidity | Oct 12, 2024 | 6 | No | No |
| Escobar 2021 | Prediction of obstetrical and fetal complications using automated electronic health record data | Oct 4, 2024 | 36 | No | No |
| Gong 2022 | Risk-factor model for postpartum hemorrhage after cesarean delivery: a retrospective study based on 3498 patients | Oct 4, 2024 | 8 | No | No |
| Guedalia 2020 | Real-time data analysis using a machine learning model significantly improves prediction of successful vaginal deliveries | Oct 12, 2024 | 32 | No | No |
| Han 2020 | A new predicting model of preeclampsia based on peripheral blood test values | Oct 12, 2024 | 17 | No | No |
| Hochman 2021 | Development and validation of a machine learning-based postpartum depression prediction model: A nationwide cohort study | Oct 12, 2024 | 69 | No | No |
| Hoffman 2021 | A machine learning algorithm for predicting maternal readmission for hypertensive disorders of pregnancy | Oct 12, 2024 | 47 | No | No |
| Houri 2022 | Prediction of Type 2 Diabetes Mellitus According to Glucose Metabolism Patterns in Pregnancy Using a Novel Machine Learning Algorithm | Oct 4, 2024 | 4 | No | No |
| Jhee 2019 | Prediction model development of late-onset preeclampsia using machine learning-based methods | Oct 12, 2024 | 159 | No | No |
| Li 2021 | Novel electronic health records applied for prediction of pre-eclampsia: Machine-learning algorithms | Oct 1, 2024 | 20 | No | No |
| Li 2022a | Improving preeclampsia risk prediction by modeling pregnancy trajectories from routinely collected electronic medical record data | Oct 12, 2024 | 26 | No | No |
| Li 2022b | Dynamic gestational week prediction model for pre-eclampsia based on ID3 algorithm | Oct 4, 2024 | 2 | No | No |
| Liao 2022 | Development and validation of prediction models for gestational diabetes treatment modality using supervised machine learning: a population-based cohort study | Oct 1, 2024 | 17 | No | No |
| Lipschuetz 2020 | Prediction of vaginal birth after cesarean deliveries using machine learning | Oct 1, 2024 | 91 | No | No |
| Liu 2022a | Machine learning-based prediction of postpartum hemorrhage after vaginal delivery: combining bleeding high risk factors and uterine contraction curve | Oct 1, 2024 | 20 | No | No |
| Liu 2022b | Development of a prediction model on preeclampsia using machine learning-based method: a retrospective cohort study in China | Oct 1, 2024 | 20 | No | No |
| Macones 2001 | Predicting outcomes of trials of labor in women attempting vaginal birth after cesarean delivery: a comparison of multivariate methods with neural networks | Oct 1, 2024 | 91 | No | No |
| Meyer 2022 | External Validation of Postpartum Hemorrhage Prediction Models Using Electronic Health Record Data | Oct 1, 2024 | 3 | No | No |
| Nagarajan 2015 | Supervised machine learning techniques for predicting the risk levels of gestational diabetes mellitus | Oct 1, 2024 | Not found (0) | No | No |
| Piekos 2022 | The effect of maternal SARS-CoV-2 infection timing on birth outcomes: a retrospective multicentre cohort study | Oct 4, 2024 | 125 | No | No |
| Qiu 2017 | Electronic Health Record Driven Prediction for Gestational Diabetes Mellitus in Early Pregnancy | Oct 1, 2024 | 73 | No | No |
| Rueangket 2022 | Predictive analytical model for ectopic pregnancy diagnosis: Statistics vs. machine learning | Oct 1, 2024 | 4 | No | No |
| Shara 2022 | Early Identification of Maternal Cardiovascular Risk Through Sourcing and Preparing Electronic Health Record Data: Machine Learning Study | Oct 3, 2024 | 2 | No | No |
| Tsur 2020 | Development and validation of a machine-learning model for prediction of shoulder dystocia | Oct 4, 2024 | 48 | No | No |
| Westcott 2022 | Prediction of Maternal Hemorrhage Using Machine Learning: Retrospective Cohort Study | Oct 3, 2024 | 12 | No | No |
| Wong 2022 | Applying Automated Machine Learning to Predict Mode of Delivery Using Ongoing Intrapartum Data in Laboring Patients | Oct 3, 2024 | 5 | No | No |
| Wu 2021 | Early Prediction of Gestational Diabetes Mellitus in the Chinese Population via Advanced Machine Learning | Oct 3, 2024 | 126 | No | No |
| Yang 2022 | Machine Learning-Based Risk Stratification for Gestational Diabetes Management | Oct 3, 2024 | 19 | No | No |
| Zhang 2021 | Development and validation of a machine learning algorithm for predicting the risk of postpartum depression among pregnant women | Oct 3, 2024 | 102 | No | No |
| Zhang 2022a | Prediction of intrahepatic cholestasis of pregnancy in the first 20 weeks of pregnancy | Oct 3, 2024 | 11 | No | No |
| Zhang 2022b | The Prediction of Preterm Birth Using Time-Series Technology-Based Machine Learning: Retrospective Cohort Study | Oct 3, 2024 | 8 | No | No |
| Zhuetlin 2022 | Improving postpartum hemorrhage risk prediction using longitudinal electronic medical records | Oct 3, 2024 | 27 | No | No |

**SUPPLEMENTARY TABLE 4: Characteristics of studies included (n=39) in the scoping review on implementation of machine learning models to predict maternal morbidity and mortality outcomes from electronic medical record data.**

| **Study** | **Years study conducted** | **Country of study** | **Study design type (cross-sectional, case control, cohort, etc.)** | **Population** | **Specific pregnancy/postpartum, neonatal outcome for which risk is predicted** | **Demographic or clinical characteristics used to generate cohort (i.e., used as eligibility criteria)** |
| --- | --- | --- | --- | --- | --- | --- |
| **Birth Outcome (n=3)** | | | | | | |
| Piekos 2022 | 2020-2021 | USA | Cohort | Pregnant patients | Primary outcome: gestational age at delivery  Secondary outcomes: stillbirth, birthweight, fetal growth percentile, SGA, and rates of common pregnancy-related conditions. | The SARS-CoV-2 positive cohort included people who had a positive SARS-CoV-2 PCR-based test during pregnancy, subdivided by trimester of infection. Pregnant patients aged 18-44 with singleton pregnancies who delivered after 140 days’ gestational age (20 weeks), who had either commercial or state-provided Medicaid insurance |
| Zhang 2022b | 2017-2020 | China | Cohort | Pregnant patients | Preterm birth | Singleton gestations who took a pregnancy test before 12 weeks and had a vaginal birth at a hospital and did not undergo IVF, cerclage placement, or have significant cardio-pulmonary complications or comorbidities |
| Abraham 2022 | Not mentioned | USA | Cohort | Pregnant patients | Preterm birth | Pregnant patients with singleton gestations and at least one delivery at a University Hospital |
| **Mode of delivery (n=4)** | | | | | | |
| Guedalia 2020 | 2003-2014 | Israel | Cohort | Pregnant patients | Vaginal birth | Pregnant patients with singleton gestations who had a live birth at term with recorded data on the labor process (e.g. cervical examinations). Those with planned cesarean deliveries or major fetal anomalies were excluded. |
| Lipschuetz 2020 | 2003-2014 | Israel | Cohort | Pregnant patients | Vaginal birth | Pregnant patients with singleton gestations who had a live birth at term.  Those with planned cesarean deliveries, a history of >2 prior cesarean deliveries, a uterine scar other than low transverse, or major fetal anomalies were excluded. |
| Macones 2001 | 1994-1998 | USA | Case control | Pregnant patients | Vaginal birth after cesarean | Cases: Pregnant patients who did not deliver vaginally after a prior cesarean (excluded unknown uterine scars and vertical cesarean. Controls: Pregnant patients who delivered vaginally after prior cesarean |
| Wong 2022 | 2013-2019 | USA | Cohort | Pregnant patients | Vaginal birth | Pregnant patients who attempted a trial of labor and had two or more cervical examinations during their inpatient labor and delivery course. |
| **Gestational Diabetes (n=7)** | | | | | | |
| Artzi 2020 | 2010-2017 | Israel | Cohort | Pregnant patients | Gestational diabetes | Pregnant patients who had a 2-step glucose tolerance test between 24-28-weeks gestation to diagnose Gestational Diabetes |
| Liao 2022 | 2007-2017 | USA | Cohort | Pregnant patients | Need for pharmacologic treatment of gestational diabetes | Pregnant patients with a diagnosis of gestational diabetes based on screening between 24-28 week; preexisting diabetes excluded |
| Nagarajan 2015 | Not mentioned | India | Retrospective (EHR review) and prospective (questionnaire and interview) | Pregnant patients | Gestational diabetes | None used for inclusion criteria |
| Qiu 2017 | 2013-2016 | China | Case Control | Pregnant patients | Gestational diabetes | Used a filtering strategy to preselect patients as our candidate samples whose EHRs data were related to GDM, excluding those of pregestational diabetes mellitus (PGDM). Removed samples and attributes with >50% missing data. |
| Wu 2021 | 2017 & 2018 | China | Cohort | Pregnant patients | Gestational diabetes. diagnostic criteria followed the IADPSG guidelines (FPG ≥ 5.1 mM, 1-h ≥ 10 mM, and/or 2-h ≥ 8.5 mM). | Women with pre-gestational diabetes (FPG ≥ 7.0 mM or glycated hemoglobin [HbA1c] ≥ 6.5%) were excluded. Samples that had a missing observation of greater than 20% were excluded from the data set. |
| Yang 2022 | 2018-2021 | UK | Cohort | Pregnant patients | High blood glucose levels | Pregnant patients with gestational diabetes and subscribed to the GDm-Health system with routine glucose reading |
| Houri 2022 | 2007-2014 | Israel | Cohort | Pregnant patients | Gestational diabetes | Eligibility was limited to women between 18 and 45 years of age with singleton gestations who delivered at a University-affiliated tertiary medical center with records of both a glucose challenge test and oral glucose tolerance test. Women who were diagnosed with gestational diabetes mellitus before 24 weeks were excluded. |
| **Cardiovascular Risks and Hypertensive Disorders of Pregnancy (n=9)** | | | | | | |
| Li 2022a | 2002-2019 | USA | Cohort | Pregnant patients | Pre-eclampsia | Pregnant patients aged 12-late 50 with complete pregnancy journeys captured in the EMR including antepartum, intrapartum, and postpartum information.  1. diagnosed with labor and delivery  2. had vaginal or cesarean section delivery  3. admission records to labor and delivery facility |
| Chen 2022 | 2012-2019 | China | Cohort | Pregnant patients | Adverse maternal outcomes: HELLP syndrome, eclampsia, cerebrovascular complications, placental abruption, acute kidney injury, pulmonary edema, liver dysfunction, disseminated intravascular coagulation, maternal death, ICU admission   Severe neonatal complications: preterm birth, fetal growth restriction/SGA, neonatal ICU admission, low Apgar scores, neonatal death | Pregnant patients > 18 years old with singleton gestations diagnosed with gestational hypertension or preeclampsia. Patients transferred from outside hospitals were excluded |
| Han 2020 | 2014-2018 | China | Case Control | Pregnant patients | Preeclampsia | Pregnant patients with preeclampsia, gestational hypertension (cases), or normal full-term pregnancies (controls). Patients with chronic kidney disease, chronic hypertension, lupus, thrombocytopenia, pre-gestational diabetes, coagulopathy, or anyone taking antiplatelets, anticoagulants, or glucocorticoids were excluded |
| Hoffman 2021 | 2015-2019 | USA | Cohort | Pregnant patients | Postpartum readmission within 42 days of delivery for hypertensive disorder of pregnancy | Pregnant patients who delivered at a large tertiary hospital |
| Jhee 2019 | 2005-2017 | South Korea | Cohort | Pregnant patients | Late-onset preeclampsia after 34-weeks gestation | Pregnant patients who delivered at a large University based health center after 24 weeks gestation |
| Li 2021 | 2016-2019 | China | Cohort | Pregnant patients | Preeclampsia | Pregnant patients who received antenatal care and delivered after 24 weeks gestation at a University-affiliated hospital branch, had complete data on all 39 features. Exclusion criteria were chronic hypertension, major fetal malformations, maternal aspirin use, or development of eclampsia, HELLP, or placental abruption |
| Liu 2022b | 2015-2019 | China | Cohort | Pregnant patients | Preeclampsia | Pregnant patients with singleton gestations resulting in a livebirth who had aneuploidy screening between 11 and 13 weeks, had complete data, and were Chinese. β-HCG and pregnancy-associated plasma protein A (PAPP-A). The sonication parameters included crown-rump length (CRL), transparent layer thickness and uterine arteries pulsatility index (UtA-PI) |
| Shara 2022 | 2017-2020 | USA | Cohort | Pregnant patients | Cardiovascular risks | Patient demographics (e.g., age, race, and geographic location), physiologic measures (e.g., blood pressure, heart rate, and oxygen saturation), symptoms (e.g., headache and shortness of breath), and health history from each patient encounter during pregnancy |
| Li 2022b | 2006-2008 & 2015-2017 | China | Cohort | Pregnant patients | Hypertensive disorders of pregnancy | Not on any oral medications. Carrying fetus "free of malformations" |
| **Cholestasis (n=1)** | | | | | | |
| Zhang 2022a | 2017-2018 | China | Case control | Pregnant patients | Intrahepatic cholestasis of pregnancy | Excluded records of heart disease, diabetes, complications during pregnancy, postpartum dysfunction etc. |
| **Mental Health (n=3)** | | | | | | |
| Amit 2021 | 2000-2017 | UK | Cohort | Postpartum patients | utilization of healthcare services: number of drug prescriptions, number of diagnoses, and number of any lab tests. age, smoking, deprivation index, pre-pregnancy BMI, ethnicity, history of premenstrual syndrome, abdominal pain, and prescription of beta blocking drugs | Non-first deliveries were not included. Included patients whose medical file was active during the pregnancy, 2 years preceding and the year after delivery. |
| Hochman 2021 | 2008-2015 | Israel | Cohort | Postpartum patients | Postpartum depression | Sociodemographic features included maternal age at birth, maternal marital status at birth, country of birth (Israel/immigrant), ethnicity (Arab, Jew), and socioeconomic status determined by primary care clinic's address (low/middle/high) |
| Zhang 2021 | 2015-2018 | USA | Cohort | Postpartum patients | Postpartum depression | Postpartum, within 1 year of childbirth |
| **Postpartum Hemorrhage (n=6)** | | | | | | |
| Liu 2022a | 2016-2020 | China | Cohort | Postpartum patients | Postpartum hemorrhage (PPH) | Cesarean deliveries, vaginal deliveries, transvaginal deliveries with uterine contraction signals; vaginal deliveries with bleeding over 500 mL, with bleeding over 1000mL as severe postpartum hemorrhage (PPH) |
| Meyer 2022 | 2019-2020 | USA | Cohort | Pregnant patients | Postpartum hemorrhage (PPH) | Excluded deliveries with gestational age less than 22 weeks, if quantitative blood loss were not documented, or if no prenatal records available |
| Westcott 2022 | 2013-2018 | USA | Cohort | Postpartum patients | Postpartum hemorrhage (PPH) | The average gestational age was 274.6 (range 107-303) days, and the average patient age was 32.7 years. 2179 (7.1%) positive hemorrhage cases observed. |
| Zheutlin 2022 | 2011-2019 | USA | Cohort | Pregnant patients | Postpartum hemorrhage (PPH) | Not described |
| Escobar 2021 | 2010-2018 | USA | Cohort | Pregnant patients | Both maternal (eg, uterine rupture, postpartum hemorrhage), fetal (eg, stillbirth), and neonatal (eg, hypoxic ischemic encephalopathy) adverse events. | Epic EMR was functioning at the hospital for 3 months; gestational age was 22 weeks on arrival; fetal heart rate was present on arrival; and delivery (live birth or fetal loss) occurred after admission. |
| Gong 2022 | 2015-2020 | China | Case Control | Pregnant patients | Postpartum hemorrhage (PPH) after Cesarean delivery (CD) | Not described |
| **Ectopic pregnancy (n=1)** | | | | | | |
| Rueangket 2022 | 2010-2022 | Thailand | Cohort | Pregnant patients | Ectopic pregnancy (EP) | Serum β-hCG level at first visit (mIU/ml), Ultrasound findings: Intra-uterine anechoic content, Endometrial thickness > 14mm, Adnexal mass of complex echogenicity, Free fluid in cul-de-sac |
| **Severe Maternal Morbidity (n=4)** | | | | | | |
| Clapp 2022b | 2016-2020 | USA | Cohort | Patients with delivery encounter | Severe Maternal Morbidity | Delivery encounters identified by ICD-10 Z37 codes. Compared model to use of the Obstetric Comorbidity Index (OB-CMI), a comorbidity-weighted risk score used during the delivery encounter to stratify a patient’s risk of SMM in clinical practice |
| Cartus 2022 | 2010-2011,& 2013–2017 | USA | Cohort | Pregnant patients | Severe Maternal Morbidity | Live, singleton deliveries occurring at Magee-Womens Hospital |
| Clapp 2022a | 2016-2020 | USA | Cross-sectional | Pregnant patients | Severe Maternal Morbidity | Racial and ethnic distribution, with more Black (13.8% vs 7.9%) and Hispanic (5.8% vs 4.6%) individuals in the Bvalid dataset; more primarily English-speaking patients (90.4% vs 86.0%; P<.001) in the Bvalid dataset; and lower comorbidity scores (median, 7 vs 11; P<.001) and rates of SMM (3.2% vs 4.2%) in the Bvalid dataset |
| Clapp 2021 | 2016-2019 | USA | Cohort | Pregnant patients | Severe Maternal Morbidity | Non-viable (defined as <23-weeks gestation) and non-liveborn (defined as 1- and 5-min Apgar scores of 0) deliveries were excluded as their delivery records are not universally completed. For women with more than one delivery during the analysis period, only the first delivery was analyzed. |
| **Shoulder Dystocia (n=1)** | | | | | | |
| Tsur 2020 | 2011-2018 | Israel | Cohort | Pregnant patients | Shoulder Dystocia | Not described |

**Supplementary Table 5: Data types, elements, and sources in studies included (n=39) in the scoping review on implementation of machine learning models to predict maternal morbidity and mortality outcomes from electronic medical record data.**

| **Study** | **Source of EMR data** | **Medical images (recordings, echotomography, ultrasounds, resonance, etc.)** | **Biological markers** | **SDOH data** | **Other data sources** | **Specify other data elements** |
| --- | --- | --- | --- | --- | --- | --- |
| **Birth Outcome (n=3)** | | | | | | |
| Piekos 2022 | Providence St Joseph Health | No | No | Yes, Race, ethnicity, insurance, education | No | NA |
| Zhang 2022b | Hangzhou Maternity and Child Health Care Hospital | Yes, Biparietal diameter, cm, Head circumference, cm, Femur length, cm, Fetal abdominal circumference, cm | No | No | No | NA |
| Abraham 2022 | Vanderbilt’s EMR database | No | Yes, linked genetic biobank | Yes, Race | Yes, Billing codes | NA |
| **Delivery Outcomes (n=4)** | | | | | | |
| Guedalia 2020 | Database of the Department of Obstetrics and Gynecology | Yes, sonographic findings, or by  Leopold maneuver | No | No | No | NA |
| Lipschuetz 2020 | Hadassah Medical Center Obstetrics Department database | No | No | No | No | NA |
| Macones 2001 | Not mentioned | No | No | No | No | NA |
| Wong 2022 | Cedars-Sinai Medical  Center, Los Angeles, California | No | No | Yes, Race/ethnicity, private insurance | No | NA |
| **Gestational Diabetes (n=7)** | | | | | | |
| Artzi 2020 | Clalit Health Services (Clalit) | No | No | No | No | NA |
| Liao 2022 | Kaiser Permanente Northern California | No | No | Yes, College education, Median house income | Yes, GDM Registry | NA |
| Nagarajan 2015 | Not mentioned | No | Yes, Hb1Ac | No | Yes, Questionnaire and direct interviews - Not mentioned which specific variables were collected through these methods and how these data were used. | NA |
| Qiu 2017 | West China Second Hospital in Chengdu,  Sichuan | No | No | No | No | NA |
| Wu 2021 | International Peace Maternal and Child  Health Hospital, Shanghai Jiao Tong University School  of Medicine | No | Yes, FPG, HbA1c, lipoprotein(a), triglyceride (TG), and apolipoprotein-B. Total 3,3,5′-triiodothyronine (TT3) | No | No | NA |
| Yang 2022 | Oxford University Hospitals | No | No | Yes, Ethnicity | No | NA |
| Houri 2022 | Not mentioned; University affiliated medical center | No | No | No | No | NA |
| **Cardiovascular Risks and Hypertensive Disorders of Pregnancy (n=9)** | | | | | | |
| Li 2022a | Mount Sinai Health System | No | No | No | No | NA |
| Chen 2022 | Tongzhou Maternal and Child  Health Care Hospital of Beijing EMR system | No | No | No | No | NA |
| Han 2020 | Fujian Maternal and Child  Health Hospital | No | No | No | No | NA |
| Hoffman 2021 | large tertiary hospital in  Newark, Delaware | No | No | No | No | NA |
| Jhee 2019 | Yonsei University  Healthcare Center | No | No | No | No | NA |
| Li 2021 | Xinhua hospital Chongming branch, affiliated to  Shanghai Jiaotong University | No | No | No | No | NA |
| Liu 2022b | First Affiliated  Hospital of Jinan University (JNU-Hospital) in Guangzhou,  China | Yes, crown-rump length (CRL),  transparent layer thickness and uterine arteries pulsatility index  (UtA-PI) | No | No | No | NA |
| Shara 2022 | Cerner EMR | No | No | Yes, Race, Zipcode | Yes, the 2 primary systems used were a direct connection to Cerner’s underlying Oracle database, as well as an enterprise data warehouse (EDW) solution | NA |
| Li 2022b | Peking University People’s  Hospital | Yes, Radial artery and fingertip volumetric pulse  waveform | Yes | No | Yes, Haidian District Maternal and Child Health Hospital, Beijing Maternity Hospital | NA |
| **Cholestasis (n=1)** | | | | | | |
| Zhang 2022a | West China Second University Hospital of Sichuan University | No | No | No | No | NA |
| **Mental Health (n=3)** | | | | | | |
| Amit 2021 | IQVIA Medical  Research Data (IMRD) | No | No | No | No | NA |
| Hochman 2021 | Clalit Health Services (CHS) EMR data warehouse | No | Yes, Blood test | Yes, Immigrant, age, marital status at childbirth, ethnicity, socioeconomic status | Yes, Clinical and obstetric | NA |
| Zhang 2021 | Weill Cornell Medicine (WCM) and NewYork-Presbyterian Hospital in New York City, USA | No | No | Yes, race, marital relationship, environment characteristics such as distance to public transportation and green space | Yes, New York City affiliated to the Patient-Centered Outcomes  Research Institute (NYC-CORN) | NA |
| **Postpartum Hemorrhage (n=6)** | | | | | | |
| Liu 2022a | First Affliated  Hospital of Jinan University (JNU-Hospital) in Guangzhou,  China | Yes, Uterine contractions measured by TOCO number (mmHG) | No | No | No | NA |
| Meyer 2022 | The University of Michigan Von Voigtlander Women’s Hospital | No | No | No | No | NA |
| Westcott 2022 | New York University Langone Health Tisch Hospital | No | No | No | No | NA |
| Zheutlin 2022 | Mount Sinai Hospital System | No | No | Yes, Race, ethnicity, insurance | No | NA |
| Escobar 2021 | EPIC | No | No | No | No | NA |
| Gong 2022 | Chongqing Medical university which includes seven medical institutions | No | No | No | No | NA |
| **Ectopic pregnancy (n=1)** | | | | | | |
| Rueangket 2022 | Phramongkutklao Hospital EMR system | Yes, Intra-uterine anechoic content, Endometrial thickness > 14mm, Adnexal mass of complex echogenicity, Free fluid in cul-de-sac | No | No | No | NA |
| **Severe Maternal Morbidity (n=4)** | | | | | | |
| Clapp 2022b | Brigham and Women’s Hospital, Boston, Massachusetts | No | No | No | No, Delivery admission history and physical notes filed in the institution’s EHR before and after the comorbidity index validation study (i.e. the training set). For the rare encounters with multiple history and physical notes (e.g., individuals with transfers between services), the note with the earliest time stamp was used. | Free text |
| Cartus 2022 | Magee Obstetric Maternal  and Infant (MOMI) database | Yes, ultrasound | No | Yes, race/ethnicity,  insurance type, age, education | No | NA |
| Clapp 2022a | EPIC | No | No | Yes, Race, primary language | No | NA |
| Clapp 2021 | EPIC | No | No | Yes, Self-identified race, payer at the time of delivery,  marital status, and a median income of patient’s zip  code, insurance | No | NA |
| **Shoulder Dystocia (n=1)** | | | | | | |
| Tsur 2020 | Sheba Medical Center | Yes, sonographic fetal biometry - fetal biparietal diameter (BPD), head circumference  (HC), abdominal circumference (AC) and femur length  (FL) | No | No | Yes, University of California San Francisco Medical Center | NA |

**Supplementary Table 6: Features used in studies included (n=39) in the scoping review on implementation of machine learning models to predict maternal morbidity and mortality outcomes from electronic medical record data.**

| **Study** | **Number of records used** | **Total number of records used for feature engineering (enter number only)** | **Total number of records used for predictive modeling (enter number only)** | **Number of features considered** | **Number of features included in final model** | **Features considered**  **(≤25 or >25)** | **Features included in final model** |  |
| --- | --- | --- | --- | --- | --- | --- | --- | --- |
| **Birth Outcome (n=3)** | | | | | | | | |
| Piekos 2022 | 882 | 705 | 177 | 24 | 24 | < 25 | Patient demographics, fetal characteristics, comorbidities, and treatment following SARS-CoV-2 infection before delivery (including encounters, diagnoses, medications, and COVID-19 severity score) |  |
| Zhang 2022b | 5187 | 4149 | 1038 | 24 | 8 | < 25 | Maternal age was the most important variable followed by triglyceride level, total bile acid level, systolic pressure during pregnancy, fundal height, uric acid level, platelet level, and pre-pregnancy weight |  |
| Abraham 2022 | 35,282 | 25,225 | 10,057 | Not mentioned | 15 | > 25 | Known fetal abnormality, Threatened premature labor, History of PTB, Hypertension Antepartum, Diabetes, Cervical shortening, Hereditary, hemolytic anemia, Hypertension, Poor fetal growth, Diabetes screen, Antenatal Screening, Pulmonary TB screen, Screen for cervix malignancy, Supervision of pregnancy, Supervision of pregnancy, Routine fetal ultrasound, Pregnancy complication, Elderly multi-gravid complication, Super. elderly mult. pregnancy |  |
| **Delivery Outcomes (n=4)** | | | | | | | | |
| Guedalia 2020 | 94,480 | 85,032 | 9448 | 47 | 47 | > 25 | Maternal parameters (9); Interpregnancy parameters (12); Index pregnancy parameters (10); Real-time intrapartum (16) |  |
| Lipschuetz 2020 | 9,888 | 6,726 | 747 | 34 | 34 | > 25 | Two different models (First-trimester model and pre-labor model) during the women's pregnancy journey. Maternal (age, gravidity, parity, previous abortions, smoking status, blood type etc.); Interpregnancy (interval, previous delivery weights, gestational age, mode of delivery etc.), index pregnancy, first cervical examination |  |
| Macones 2001 | 400 | 240 | 160 | 8 | 8 | < 25 | Substance abuse, parity, prior successful VBAC, weight gain during pregnancy, pre-pregnancy body mass index, years since last delivery, cervical dilatation on admission, and the need for labor augmentation |  |
| Wong 2022 | 29,068 | 30,346 | 7,586 | Not mentioned | Not mentioned | > 25 | Age, Race/ethnicity, Private insurance, Nullipara, Previous cesarean, Body mass index, Gestational age (wk), Cervical dilatation at admission (cm), Epidural, Induction methods. Type of labor (e.g., spontaneous vs. induced), membrane status, and baseline cervical examination. In addition, intrapartum data were collected which included iterative information obtained throughout the labor course: vital signs, pain scores, cervical examinations, fetal heart rate assessments, and medications |  |
| **Gestational Diabetes (n=7)** | | | | | | | | |
| Artzi 2020 | 588,622 | 451,402 | 137,220 | 2,355 | 9 | > 25 | (1) What is your date of birth?  (2) What are your weight and height?  (3) How many of your first-degree relatives have diabetes?  (4) Has a doctor ever told you that you have  (a) High cholesterol? (b) Had a miscarriage? (c) PCOS? (d) Pre-diabetes? (e) Heart disease? (f) GDM? (g) High BP?  (5) If you had a HbA1c% test, what was the highest value recorded?  (6) Have you given birth before?  (7) How many times?  (8) During your previous pregnancy, did you  undergo GCT or OGTT?  (9) What were the results? |  |
| Liao 2022 | 30474 | 27,420 | 3,234 | 176 | 176 | > 25 | Meeting glycemic control goal, fasting; week of GDM diagnosis, OGTT, fasting value, per 1mg/dL increase; No. of fasting SMBG per week; history of GDM; GDM diagnosed by C-C; No. of 1-h post-dinner SMBG per week; pre-pregnancy exercise <150 minutes per week; meeting glycemic control goal, 1h post dinner; Diastolic BP per 1mmHG increase |  |
| Nagarajan 2015 | 1019 | 712 | 307 | Not mentioned | 8 | > 25 | Plasma glucose concentration from oral glucose tolerance test  Hb1Ac (Glycated hemoglobin)  Pedigree (Family History Details)  Body Mass Index  Previous history of gestational diabetes mellitus  Age of patients  Lifestyle (sedentary/normal)  Number of previous pregnancies |  |
| Qiu 2017 | 4,378 | 3940 | 438 | 50 | 50 | > 25 | 50 features (this is approximately 10% of the attributes available from EMR data)  High risk, Marriage years, Height, Pregnancy times, Age of pregnant patient and husband, BMI, lab values |  |
| Wu 2021 | A total of 16,819 and 14,992 cases were included in the training and testing sets, respectively | 16,819 | 15,371 | 17 | 7 | > 25 | Age, Previous GDM, Family history of diabetes in a first-degree relative, Multiple pregnancy, FPG, HBA1c, Triglyceride |  |
| Yang 2022 | 1,148 pregnancies (272,712 blood glucose readings) | 4573 | 1192 | Not mentioned | Not mentioned | < 25 | Age, BMI, gestational day, ethnicity, blood glucose measurements, medications |  |
| Houri 2022 | 6092 | 4264 | 1828 | 10 | 10 | < 25 | Maternal age, parity, gravidity, gestational  age at delivery, 50 g GCT and 100 g OGTT results and neonatal birthweight |  |
| **Cardiovascular Risks and Hypertensive Disorders of Pregnancy (n=9)** | | | | | | | | |
| Li 2022a | 114,312 | 60,879 | 47,678 | 2989 - 3294 for 17 time  points in antepartum, 4136 for intrapartum, and 5391 for postpartum | 148 features | > 25 | >25: An example - CBC-related characteristics dominated in antepartum; pregnancy complications associated with intrapartum; follow-up care impacted postpartum. |  |
| Chen 2022 | 1829 | 1647 | 182 | Maternal: 77  Neonatal: 80 | at least 7 features  Model with 10 features had highest AUC | > 25 | Maternal: platelet count, fetal head/abdominal circumference ratio, gestational age at diagnosis, low plateletcrit (categorical), plateletcrit, 24 h urine protein, creatinine, high serum chlorine (categorical), fetal femur length/abdominal circumference ratio, and prothrombin time.  Neonatal: gestational age at diagnosis, fetal femur length, fetal head/abdominal circumference ratio, fetal biparietal diameter, fetal head circumference, 24 h urine protein, abnormal fetal head circumference (categorical), umbilical artery blood flow, resistance index, and fetal pulsatility index |  |
| Han 2020 | 568 | 379 | 189 | 25 | 5 | < 25 | 5 factors with the greatest influence on preeclampsia are ALB, MPV, BUN, LDH  and TG |  |
| Hoffman 2021 | 25559 | 23307 | 2590 | Not mentioned | 31 | > 25 | African American race, Systolic BP EMA change after delivery, patient flagged high risk of HTN, ketorolac administered, shock EMA change, diastolic BP after delivery, weight gain (lb), score computed from respiratory-related drugs, patient flagged high risk of preeclampsia, diastolic BP EMA change, score computed from vital signs after delivery, score based on improvement of vital signs after delivery, patient flagged risk of hypertension, diastolic BP EMA after delivery, last ALT level, mean AST level, magnesium sulfate administered, respiratory score, shock EMA score, score computed from proteinuria diagnostic criteria, score based on liver laboratory exams, score computed from narcotic drugs administered ,score based on history of hypertension, score based on infections, vital trend score, score based on antibiotics, score based on clotting drugs and hemorrhage diagnoses, hypertension drug score |  |
| Jhee 2019 | 11,006 | 7,704 | 3,302 | 25 | 7 | > 25 | Systolic blood pressure, serum blood urea nitrogen and creatinine levels, platelet counts, serum potassium level, white blood cell count, serum calcium level, and urinary protein |  |
| Li 2021 | 5,243 | 5,052 | 191 | 39 | 39 | > 25 | Demographics, pregnancy history, medical conditions outside of pregnancy, and lab data. |  |
| Liu 2022b | 11,152 | 10,037 | 1,115 | 18 | 18 | < 25 | Demographic characteristics (age, height, weight, smoking history), parity, method of conception, gestational age, previous diagnosis of hypertension, systemic lupus erythematosus (SLE), or antiphospholipid syndrome (APS), history of DM, GDM or PE, MAP, Beta-HCG, PAPP-A, pulsation index of bilateral uterine arteries |  |
| Shara 2022 | 32,409 | 32409? | 32409? | 24 | NA | < 25 | Symptoms (variable risks)  • Dyspnea (red flag risk)  • Orthopnea (red flag risk)  • Tachypnea  • Asthma unresponsive to therapy  • Swelling in face or hands  • New or worsening headache  • Heart palpitations  • Dizziness or syncope  • Chest pain  Physical findings (variable risks)  • Loud heart murmur  • Basilar crackles in lungs  • Resting heart rate≥120 beats per minute (red flag risk)≥110 beats per minute  • Systolic blood pressure ≥160 mm Hg (red flag risk)≥140 mm Hg  • Respiratory rate ≥30 (red flag risk)≥24  • Oxygen saturation ≤94% (red flag risk)≤96%  Medical history (static risks)  • Aged ≥40 years  • Race=African American  • Pre-pregnancy obesity (BMI≥35)  • Pre-pregnancy diagnosis of diabetes  • Pre-pregnancy diagnosis of hypertension  • Substance use (nicotine, cocaine, alcohol, and methamphetamines)  • History of chemotherapy  • History of complications in labor or delivery  • History of heart disease |  |
| Li 2022b | 932 pregnant women with 1818 tests | 512 | 220 | 32 | 24 | > 25 | Static: multiple births, spontaneous miscarriage history, history of hypertension in pregnancy, history of diabetes mellitus, family history of hypertension preconception BMI. Dynamic: gestational week, BMI during pregnancy, systolic BP, diastolic BP, mean arterial pressure, waveform area parameters cardiac output, cardiax index, total peripheral resistance, hematocrit, mean platelet volume, platelets, ALT, AST, creatinine, uric acid, PIGF, pulse pressure |  |
| **Cholestasis (n=1)** | | | | | | | | |
| Zhang 2022a | 428 | 365 | 122 | Seven maternal characteristics indices for analysis and forty-three routine blood examination indices were obtained from routine hepatic, renal, and coagulation function examinations | 15 | > 25 | Maternal characteristics indices (age, parity, gestational age, BMI, number of fetuses, maternal complications, pregnancy outcome), ALT, MCHC, r-GT, APTT, Fg, P-LCR, lactate dehydrogenase (LDH), and creatinine (Cr) levels |  |
| **Mental Health (n=3)** | | | | | | | | |
| Amit 2021 | 266,544 | 260,585 | 5,959 | unsure | 69 | > 25 | (1) Demographic, socio- economic and personal measures (age, ethnicity, marital status, deprivation index, pre-pregnancy BMI, habits of smoking, alcohol use and drug use); (2) Medical diagnoses during pregnancy (mental disorders and symptoms, pregnancy complications, other relevant health conditions; (3) Labor complications (cesarean section, episiotomy) and infant-related measures (gestational week, birth weight, APGAR score); (4) History of medical diagnoses within 2  years before the pregnancy; (5) Drug prescriptions during and prior-to pregnancy (antidepressants, antibacterials, antihistamines, beta-blocking agents); (6) Healthcare utilization, measured by counts of visits, diagnoses and drug prescriptions during and prior-to pregnancy. |  |
| Hochman 2021 | 214,359 | 185,029 | 29,330 | 156 | Not mentioned | > 25 | Sociodemographic, clinical, and obstetric (gestational and delivery) |  |
| Zhang 2021 | 15,197 | 12,157 | 3,040 | 32 | 32 | > 25 | demographic, health service utilization, mental health history, newly diagnosed mental health conditions during pregnancy, other obstetric diagnoses during pregnancy and vital signs. |  |
| **Postpartum Hemorrhage (n=6)** | | | | | | | | |
| Liu 2022a | 7565 cesarean deliveries and 15,809 vaginal deliveries; 10520 transvaginal with uterine contraction signals | 9468 | 1052 | 49 | 49 | > 25 | All assessment table features, rating scale features, and uterine contractions readings |  |
| Meyer 2022 | 5,261 | 4,735 | 526 | 58 | 58 | > 25 | Admission intake, intra-pregnancy, pre-pregnancy, delivery encounter |  |
| Westcott 2022 | 30,867 | 21,606 | 9261 | 497 | 28 | > 25 | body mass index, admission hematocrit, cesarean delivery prior to labor or rupture, scheduling status of cesarean delivery, and admission platelet count. |  |
| Zheutlin 2022 | 70,948 | 56,713 | 14,235 | 80 | 24 | > 25 | Anemia, Mean corp. hemoglobin, RBC distribution, Min SBP, Min DBP, Min absolute neutrophils, Min WBC, Min platelets, Multiple gestation, Gestation weeks at admission, temperature, Prior PPH, Max SBP, Max pulse antepartum, Hemoglobin test frequency, Bupivacaine total dose, Min hemoglobin, Max platelets, Magnesium given in hospital, Assisted reproductive tech |  |
| Escobar 2021 | 239,526 | 191620 | 47906 | Not mentioned | 35 | > 25 | Vital signs, Maternal features, laboratory results, admission and discharge date/time, age, gestational age in weeks and days, multiple gestation, body mass index (BMI), presence of gestational diabetes or diabetes mellitus, time of rupture of membranes, amniotic fluid characteristics, use of intrauterine fetal monitoring, history of emboli, history of hypertension, and the delivery date/time. Repeating variables with their date/time stamps: vital signs, pulse oximetry, neurologic status,20 station of fetal descent, cervical dilation, and multiple laboratory tests, Comorbidity Point Score version 2 (COPS2) and Acute Physiology Score, version 2 (LAPS2) |  |
| Gong 2022 | 3498 | 2448 | 1050 | 56 | 46 | > 25 | Clinical information of the patients, including laboratory examination records, imaging examination records, diagnosis and treatment process |  |
| **Ectopic pregnancy (n=1)** | | | | | | | | |
| Rueangket 2022 | 407 | 306 | 101 | 22 | 22 | < 25 | Age group (years), BMI (kg/m2), Parity, Gestational age at diagnosis (days), History of pelvic surgery, Smoking, History of pelvic inflammatory disease (PID), Current use of emergency pill, Assisted reproductive technology, Abdominal pain, Abnormal vaginal bleeding, Abnormal vaginal bleeding, Nausea, vomiting, Fainting, Abdominal tenderness, Cervical motion tenderness, Serum marker: Serum β-hCG level at first visit (mIU/ml), Ultrasound findings: Intra-uterine anechoic content, Endometrial thickness > 14mm, Adnexal mass of complex echogenicity, Free fluid in cul-de-sac |  |
| **Severe Maternal Morbidity (n=4)** | | | | | | | | |
| Clapp 2022b | 19,794 | 15760 | 4034 | NA | NA | NA | bag-of-words NLP model using monograms (single words) |  |
| Cartus 2022 | 19,266 (years: 2010-2011) and  47,067 (years: 2013-2017) | 677 | 497 | ~53 | Used different features for the 7 different models | > 25 | Maternal demographic  and behavioral variables  Maternal health  variables  Labor and delivery  variables  Fetal and infant  variables |  |
| Clapp 2022a | 13,572 | 10,250 | 3,322 | NA | NA | NA | Monogram of words and frequency of words from clinical notes. Only  words that occurred in 5% but 80% of notes were included |  |
| Clapp 2021 | 17,835 | 11816 | 6019 | 140 | 9 | > 25 | ICD codes for SMM:  D50 Iron deficiency anemia  D68 Other coagulation defects \  N97 Female infertility  O30 Multiple gestation  O34 Maternal care for  abnormality of pelvic organs  O43 Placental disorders  O44 Placenta previa  R79 Other abnormal findings of  blood chemistry  Z98 Other postprocedural states |  |
| **Shoulder Dystocia (n=1)** | | | | | | | | |
| Tsur 2020 | 686 singleton  vaginal deliveries, of which 131 were complicated by ShD, and the validation cohort included 2584 deliveries, of which 31 were complicated by ShD | 53754 | 23794 | unsure | 18 | < 25 | Maternal age, gestational age (GA) at delivery, maternal height, maternal weight, smoking status, gravidity, parity, intrauterine fetal death, ShD in a previous pregnancy, pregestational diabetes, gestational diabetes, insulin treatment, fetal sex, four sonographic biometric measurements (fetal biparietal diameter (BPD), head circumference (HC), abdominal circumference (AC) and femur length (FL)) and the EFW |  |

**Supplementary Table 7: Feature selection methods used in studies included (n=39) in the scoping review on implementation of machine learning models to predict maternal morbidity and mortality outcomes from electronic medical record data.**

| **Study** | **Whether feature selection done or not (Yes/No)** | **Feature Selection methods used (e.g., Rankers, Wrappers, other; Rankers includes Correlation, Mutual Information, others)** | **Whether feature construction done or not (Yes/No)** | **Type of feature construction/engineering (Simple/Complex)** | **Simple feature construction/engineering methods used (log, normalization, PCA, SVD etc.)** | **Complex feature construction methods used (includes Embeddings by DNN, as well as Clustering, Latent Dirichlet Allocation, other latent variable methods, and Predicate Invention)** |
| --- | --- | --- | --- | --- | --- | --- |
| **Birth outcome (n=3)** | | | | | | |
| Piekos 2022 | Yes | Propensity score matching, nearest neighbors | Yes | Simple | Median imputation was used for missing values | Not mentioned |
| Zhang 2022b | No | Not mentioned | No | Not mentioned | Not mentioned | Not mentioned |
| Abraham 2022 | No | Not mentioned | Yes | Complex | Not mentioned | NLP, one-hot encoding, clustering |
| **Mode of delivery (n=4)** | | | | | | |
| Guedalia 2020 | No | Not mentioned | No | Not mentioned | Not mentioned | Not mentioned |
| Lipschuetz 2020 | No | Not mentioned | Yes | Simple | Median imputation was applied for missing values | Not mentioned |
| Macones 2001 | No | Not mentioned | No | Not mentioned | Not mentioned | Not mentioned |
| Wong 2022 | No | Not mentioned | Yes | Simple | Median imputation was used for missing values | Not mentioned |
| **Gestational Diabetes (n=7)** | | | | | | |
| Artzi 2020 | Yes | Not mentioned | Yes | Not mentioned | Not mentioned | Not mentioned |
| Liao 2022 | Yes | Manual selection - based on risk factors associated with GDM treatment modality and input from clinicians | Yes | Simple | Random forest algorithm was used for missing values | Not mentioned |
| Nagarajan 2015 | No | Not mentioned | No | Not mentioned | Not mentioned | Not mentioned |
| Qiu 2017 | Yes | Embedded feature selection method | Yes | Simple | Discretization, max-min normalization was applied. kNN interpolation algorithm was used for missing value analysis. | Not mentioned |
| Wu 2021 | Yes | Correlation, sequential forward feature selection method, variable-way hierarchical clustering results using distance metrics based on Spearman correlation coefficients | Yes | Simple | Missing value analysis | Not mentioned |
| Yang 2022 | No | Not mentioned | Yes | Simple | Missing value analysis | Not mentioned |
| Houri 2022 | No | Not mentioned | No | Not mentioned | Not mentioned | Not mentioned |
| **Cardiovascular Risks and Hypertensive Disorders of Pregnancy (n=9)** | | | | | | |
| Li 2022a | Yes | Tree-based models (XGB, random forest), penalized logistic regression with the adaptive LASSO, and univariate analysis | Yes | Simple | Functional principal component analysis | Not mentioned |
| Chen 2022 | Yes | The predictor variables were filtered using univariate analysis and backward feature elimination/selection methods and applied in the two selected models for clinical application | Yes | Simple | k-nearest neighbor algorithm was used for missing value analysis | Not mentioned |
| Han 2020 | Yes | The correlation of the influencing factors was determined according to the magnitude of the weights | No | Not mentioned | Not mentioned | Not mentioned |
| Hoffman 2021 | Yes | Information gain and correlation | Yes | Simple | Data imputation technique used for missing value analysis. Variables were transformed based on their distribution or correlation with complication. | Not mentioned |
| Jhee 2019 | Yes | Pattern recognition and cluster analysis were used for repeated measured data, and subsequently, sequential polynomial regression analysis, cluster analysis by the k-means algorithm, and odds ratio were used to assess the pattern change in variables. | Yes | Simple | Data imputation was applied for missing data | Not mentioned |
| Li 2021 | Yes | Manual chart review was done to identify and verify the features that may modify the risk of pre-eclampsia | Yes | Simple | One-hot encoder, ordinal encoder, standardization | Not mentioned |
| Liu 2022b | No | Not mentioned | Yes | Simple | Data standardization | Not mentioned |
| Shara 2022 | No | Not mentioned | Yes | Simple | Concatenation, anonymization, standardization and normalization. | Not mentioned |
| Li 2022b | Yes | The odds ratio, independent sample t test, and parameter list were finalized based on actual needs and related studies | No | Not mentioned | Not mentioned | Not mentioned |
| **Cholestasis (n=1)** | | | | | | |
| Zhang 2022a | Yes | Least absolute shrinkage and selection operator (LASSO) regression was applied for variable selection. | No | Not mentioned | Not mentioned | Not mentioned |
| **Mental Health (n=3)** | | | | | | |
| Amit 2021 | Yes | Not mentioned | Yes | Simple | Mean imputation was used for missing values of continuous variables, one-hot encoding was performed for categorical variables. | Not mentioned |
| Hochman 2021 | No | Not mentioned | No | Not mentioned | Not mentioned | Not mentioned |
| Zhang 2021 | Yes | Sequential forward selection along with clinicians/expert adjudicated feature selection approach | Yes | Simple | NLP regular expression, normalization, dummy encoding, mean imputation for missing values | Not mentioned |
| **Postpartum Hemorrhage (n=6)** | | | | | | |
| Liu 2022a | No | Not mentioned | No | Not mentioned | Not mentioned | Not mentioned |
| Meyer 2022 | No | Not mentioned | Yes | Complex | Not mentioned | Bagged tree models used to non-parametrically impute missing values for continuous variables |
| Westcott 2022 | Yes | Recursive feature selection, selection by filtering, observing feature importance, and domain knowledge | No | Not mentioned | Not mentioned | Not mentioned |
| Zheutlin 2022 | Yes | Gradient boosting, adaptive lasso regression, and logistic regression | Yes | Not mentioned | Functional principal components analysis, normalization | Not mentioned |
| Escobar 2021 | Yes | LASSO, gradient boosting, estimation of relative contribution, and clinical judgment. | Yes | Not mentioned | Not mentioned | Not mentioned |
| Gong 2022 | Yes | LASSO model was used to finalize the list of variables to be used in ML model | Yes | Simple | Miss-forest algorithm was used for missing value analysis, propensity score matching (PSM) | Not mentioned |
| **Ectopic pregnancy (n=1)** | | | | | | |
| Rueangket 2022 | Yes | Naive Bayes algorithm, correlation, forward/backward stepwise feature selection | Yes | Simple | k-NN based algorithm used for data imputation. | Not mentioned |
| **Severe Maternal Morbidity (n=4)** | | | | | | |
| Clapp 2022b | No | Not mentioned | Yes | Simple | Features constructed using standard text mining approaches. | Not mentioned |
| Cartus 2022 | Yes | Expert opinion | Yes | Simple | Mode and mean imputation for missing value analysis, standardization, log transformation, Box-Cox transformation. | Not mentioned |
| Clapp 2022a | No | Not mentioned | Yes | Complex | Not mentioned | Bag-of-words/NLP model |
| Clapp 2021 | Yes | Traditional logistic regression, least absolute shrinkage selection operator (LASSO) for variable selection were compared | Yes | Simple | Log transformation | Not mentioned |
| **Shoulder Dystocia (n=1)** | | | | | | |
| Tsur 2020 | No | Not mentioned | Yes | Simple | Standardization (centered and scaled to mean zero with a SD of 1 (z-score)) | Not mentioned |

**Supplementary Table 8: Machine learning methods used in studies included (n=39) in the scoping review on implementation of machine learning models to predict maternal morbidity and mortality outcomes from electronic medical record data.**

*Methods shorthand*

RF: Random Forest; Boosting: Boosting Methods; LR: Logistic Regression; RR: Ridge Regression; SVM: Support Vector Machine; NN: Neural Network; KNN: K-Nearest Neighbor; DT: Decision Tree; LASSO: Least Absolute Shrinkage and Selection Operator; CART: Classification and Regression Tree; LinR: Linear Regression; GLM: Generalized Linear Model; EN: Elastic Net; SL: Super Learner

*Performance metrics shorthand*

AUC: area under the receiver operating characteristic (ROC) curve; PR-AUC: area under the precision recall (PR) curve; sensitivity/recall/true positive rate (TPR); specificity/true negative rate (TNR); precision/positive predictive values (PPV); FPR: false positive rate; NPV: ​ negative predictive values; RMSE: root mean square deviation/error; MSE: mean squared error; MAE: mean absolute error.

| **Study** | **ML methods tested for prediction model** | **ML methods finally selected (i.e., one with best performance)** | **Were data labeled by computable phenotype (Yes/No)** | **Validation techniques used** | **Performance metrics used** | **Were confidence intervals or significance results on performance metrics calculated? (Yes/No)** | **Software/tools used** |  |
| --- | --- | --- | --- | --- | --- | --- | --- | --- |
| **Birth Outcome (n=3)** | | | | | | | | |
| Piekos 2022 | LinR, Boosting, RF, RR | RF | Yes | training and testing sets | accuracy, R2, RMSE | Yes (p-value) | Python, R |  |
| Zhang 2022b | Boosting, NN | NN | Yes | training and testing sets, 5-fold cross validation | AUC, accuracy, sensitivity, specificity, | No | Python |  |
| Abraham 2022 | Boosting, LR | Boosting | Yes | training and testing sets, validation set | AUC, PR-AUC | No | Python |  |
| **Delivery Outcomes (n=4)** | | | | | | | | |
| Guedalia 2020 | Boosting | Boosting | No | cross validation | AUC, sensitivity, specificity, PPV, NPV | Yes (CI for AUC, p-value for AUC comparison) | Python, Excel, SPSS |  |
| Lipschuetz 2020 | Boosting | Boosting | No | 10-fold cross validation | AUC | Yes (CI for AUC) | Python, Excel, SPSS |  |
| Macones 2001 | NN | NN | No | training and testing sets | sensitivity, specificity, accuracy, AUC | No | BrainMaker |  |
| Wong 2022 | RF, Boosting, SVM, EN | RF | Yes | 5-fold cross validation | AUC, PR-AUC, Brier score | No | SPSS |  |
| **Gestational Diabetes (n=7)** | | | | | | | | |
| Artzi 2020 | Boosting | Boosting | Yes | validation set | AUC, PR-AUC | Yes (CI for AUC and PR-AUC) | Not mentioned |  |
| Liao 2022 | LR, CART, LASSO, SL with RF and Boosting | SL with RF and Boosting | No | validation set, 10- fold cross validation | AUC | Yes (CI for AUC) | Not mentioned |  |
| Nagarajan 2015 | PDC (potential diabetes classifier)              (self-developed method, basically decision tree) | PDC | Not mentioned | Not mentioned | accuracy, precision, recall | No | ROSE2 system |  |
| Qiu 2017 | Cost-sensitive Hybrid Model (CSHM), LR, Bayesian network, NN, SVM, DT | CSHM | Yes | 10-fold cross validation | accuracy, AUC, TPR, FPR | Yes (confidence analysis on page 10; p-value) | R |  |
| Wu 2021 | LR, KNN, SVM, NN | NN, LR | Yes | training and testing sets, 10-fold cross validation | AUC, decision curve analysis (DCA) | Yes (p-value) | MATLAB |  |
| Yang 2022 | LinR, RF, Boosting | Boosting | Yes | training and testing sets, validation set | MSE, R2, MAE, accuracy | Yes (CI for MSE, R2, MAE and accuracy; p-value) | Python |  |
| Houri 2022 | Boosting, LR | Boosting | Yes | training and testing sets | AUC, accuracy, specificity, sensitivity, PPV, NPV | Yes (p-value) | SAS, Python |  |
| **Cardiovascular Risks and Hypertensive Disorders of Pregnancy (n=9)** | | | | | | | | |
| Li 2022a | Boosting | Boosting | Yes | cross validation: validation set | AUC, specificity, sensitivity, PPV, NPV | No | Python, R |  |
| Chen 2022 | RF, CART, DT, Boosting, KNN, NN, LR, GLM, EN, naïve Bayesian, flexible discriminant analysis (FDA),nearest shrunken centroids, bagged MARS | RF, Boosting | Yes | 10-fold cross validation | AUC, sensitivity, specificity | Yes (CI for AUC, sensitivity and specificity) | R |  |
| Han 2020 | NN, LR | NN | Yes | training and test sets | accuracy | No | SPSS, Python, R |  |
| Hoffman 2021 | DT, LR, Boosting | Boosting | Yes | validation set, 10-fold cross validation | AUC, PPV, NPV, precision, recall | No | Python |  |
| Jhee 2019 | DT, SVM, RF, Boosting, LR, naïve Bayesian | Boosting | Yes | validation set | AUC, accuracy, sensitivity, specificity, detection rate, calibration plot | No | R |  |
| Li 2021 | LR, RF, SVM,  Boosting | Boosting | Yes | 5-fold cross validation, validation set | AUC, accuracy, precision, recall, FNR, F1 score, Brier score | No | Python, R |  |
| Liu 2022b | NN, LR, SVM, DT, RF | RF | Yes | 10-fold cross validation | AUC, Brier score, precision, accuracy, recall, F1 score | Yes (CI for AUC, precision, recall rate, and Brier score; p-value) | SPSS, Python |  |
| Shara 2022 | HOPE-CAT (self developed method from previous papers) | Not mentioned | Not mentioned | Not mentioned | Not mentioned | No | 4 Cerner Millennium electronic medical record software, PeriBirth, R, Microsoft Azure Cloud, Microsoft Azure Data Studio, Microsoft Azure Machine Learning Studio, virtual machine, Microsoft SQL Server Management Studio, Invaryant’s health platform, HOPE-CAT. |  |
| Li 2022b | DT | DT | Not mentioned | training and testing sets | precision, recall, F1 score, accuracy | No | SPSS, Python |  |
| **Cholestasis (n=1)** | | | | | | | | |
| Zhang 2022a | CART, LR, Boosting, RF | Boosting | Yes | training and testing sets, k-fold cross validation | accuracy, sensitivity, specificity, AUC | Yes (CI for sensitivity, specificity) | SPSS, Python |  |
| **Mental Health (n=3)** | | | | | | | | |
| Amit 2021 | Boosting | Boosting | Yes | cross validation, validation set | AUC, sensitivity, specificity | Yes (CI for AUC; p-value for AUC comparison; sensitivity specificity with CI) | Not mentioned |  |
| Hochman 2021 | Boosting | Boosting | Yes | training and testing sets, validation set | AUC, PPV, NPV, sensitivity, specificity, calibration plot | Yes (CI for AUC by bootstrapping) | R, Python |  |
| Zhang 2021 | RF, DT, Boosting, LR, multilayer perceptron (MLP) | LR | Yes | 5-fold cross validation, validation set | AUC, sensitivity, specificity, Brier score, PPV, NPV | Yes (CI for AUC) | Python |  |
| **Postpartum Hemorrhage (n=6)** | | | | | | | | |
| Liu 2022a | LR, Boosting, RF, KNN | Boosting, LR | Yes | cross validation | AUC, Brier score, decision curve, sensitivity, specificity | Yes (CI for AUC) | Python |  |
| Meyer 2022 | LR, LASSO, RF, Boosting | RF, Boosting | Yes | 10-fold cross validation | AUC, calibration plot | Yes (CI for AUC; p-value) | R |  |
| Westcott 2022 | LR, RF, Boosting, SVM | Boosting | Yes | training and testing sets, validation set | accuracy, AUC, sensitivity | Yes (CI for AUC) | R |  |
| Zheutlin 2022 | Boosting | Boosting | Yes | training and testing sets, 10-fold cross validation | sensitivity, specificity, PPV, NPV, AUC | Yes (CI for all performance metrics using bootstrap) | Python |  |
| Escobar 2021 | LR, Boosting, LASSO, RF | LR | Yes | training and testing sets, validation set | AUC, sensitivity, specificity, PPV | No | Not mentioned |  |
| Gong 2022 | LR, Boosting, RF, CART, NN | RF | Not mentioned | training and testing sets | specificity, precision, recall, F1 score, AUC | Yes (CI for AUC; p-value) | R, SPSS |  |
| **Ectopic pregnancy (n=1)** | | | | | | | | |
| Rueangket 2022 | LR, SVM, NN, DT | LR | Yes | 5-fold cross validation | AUC, sensitivity, specificity | Yes (CI for AUC, sensitivity, specificity) | RapidMiner |  |
| **Severe Maternal Morbidity (n=4)** | | | | | | | | |
| Clapp 2022b | Bag-of-Words | Bag-of-Words | No | 10-fold cross validation; human specialists review | AUC, sensitivity, PPV | Yes (CI for AUC, p-value for AUC comparison) | R, Stata |  |
| Cartus 2022 | SL | SL | Yes | 10-fold cross validation, training and testing sets | AUC, PR-AUC,  accuracy, sensitivity, specificity, PPV, NPV, detection rate | No | R |  |
| Clapp 2022a | Bag-of-Words, LASSO | Bag-of-Words | Yes | validation set, training and testing sets | AUC, calibration plots | Yes (CI for AUC; p-value) | R, Stata |  |
| Clapp 2021 | LR, LASSO, RR, EN | LASSO | Yes | cross validation, validation set, training and testing sets | AUC, Hosmer–Lemeshow tests | Yes (CI for AUC; p-value) | Stata |  |
| **Shoulder Dystocia (n=1)** | | | | | | | | |
| Tsur 2020 | LASSO | LASSO | Yes | training and testing sets, 10- fold cross validation, validation set | AUC | Yes (CI for AUC; p-value) | R |  |
